# Supplementary material for: Noncanonical usage of stop codons in ciliates expands proteins with structurally flexible Q-rich motifs
Source: eLife. 2024 Feb 23;12:RP91405. doi: 10.7554/eLife.91405 (PMC10942620; doi:10.7554/eLife.91405)
Supplement: Figure 1—source data 1. [file elife-91405-fig1-data1.zip › Figure1/Figure1_source_data_labelled.pdf]

Figure 1

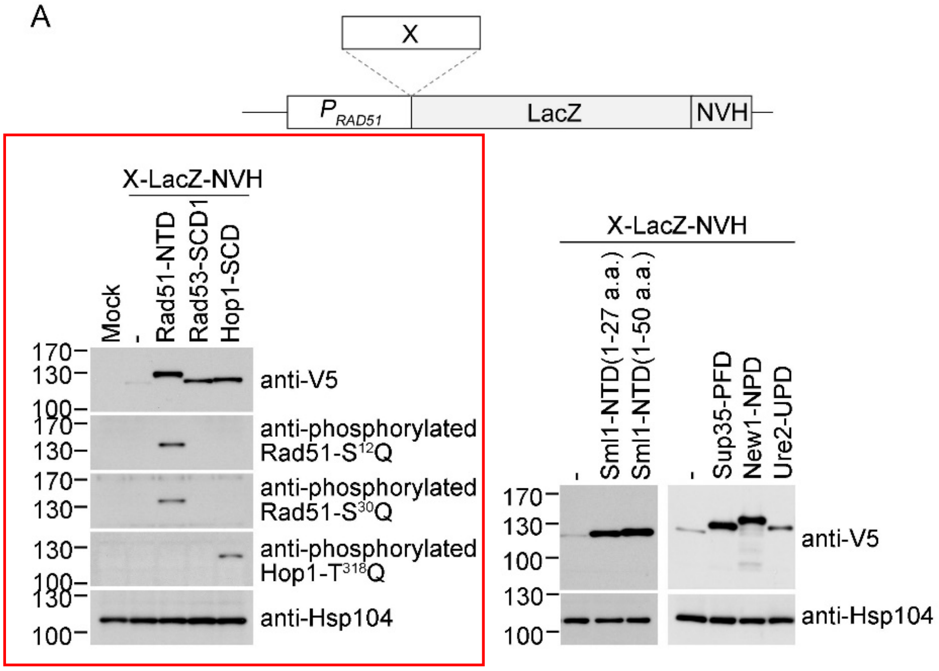
A
X
P_{RAD51}
LacZ
NVH
X-LacZ-NVH
Mock
-
Rad51-NTD
Rad53-SCD1
Hop1-SCD
anti-V5
anti-phosphorylated Rad51-S12Q
anti-phosphorylated Rad51-S30Q
anti-phosphorylated Hop1-T318Q
anti-Hsp104
Sml1-NTD(1-27 a.a.)
Sml1-NTD(1-50 a.a.)
Sup35-PFD
New1-NPD
Ure2-UPD
170
130
100

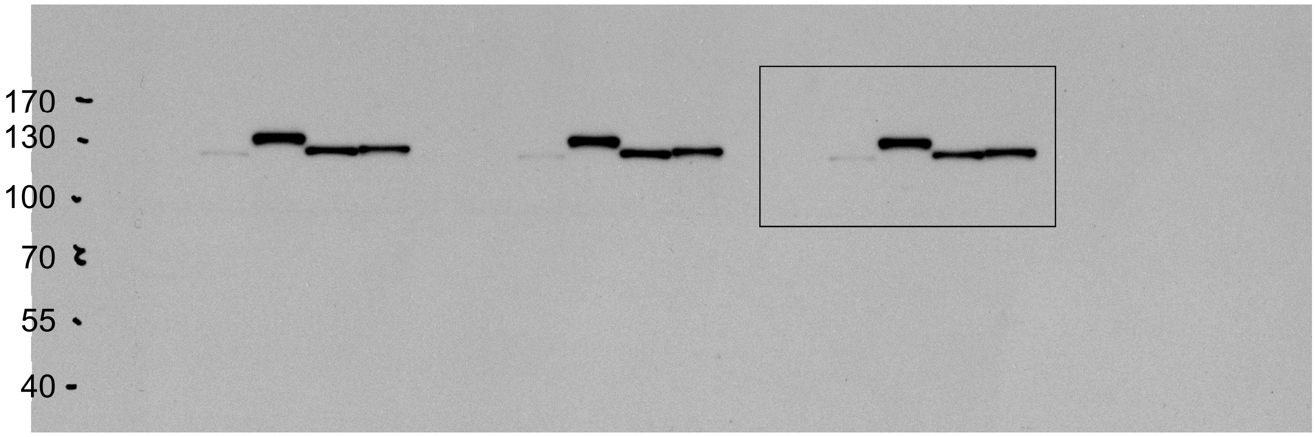
anti-V5
170
130
100
70
55
40

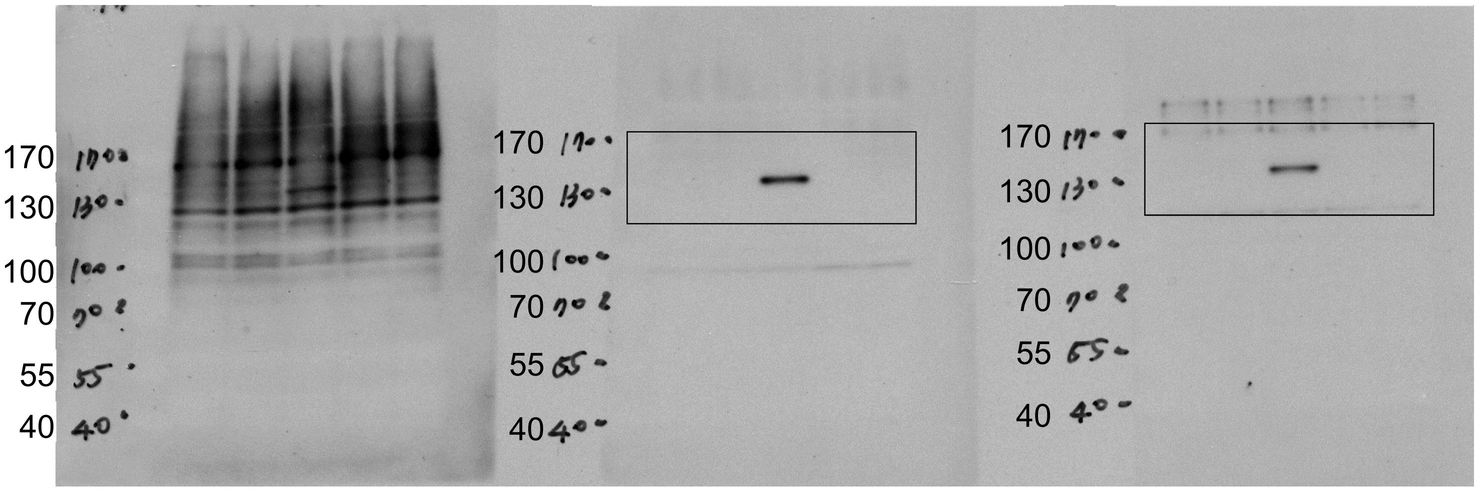
anti-phosphorylated Rad51-S12Q
anti-phosphorylated Rad51-S30Q
170
130
100
70
55
40

anti-phosphorylated Hop1-T^{318}Q

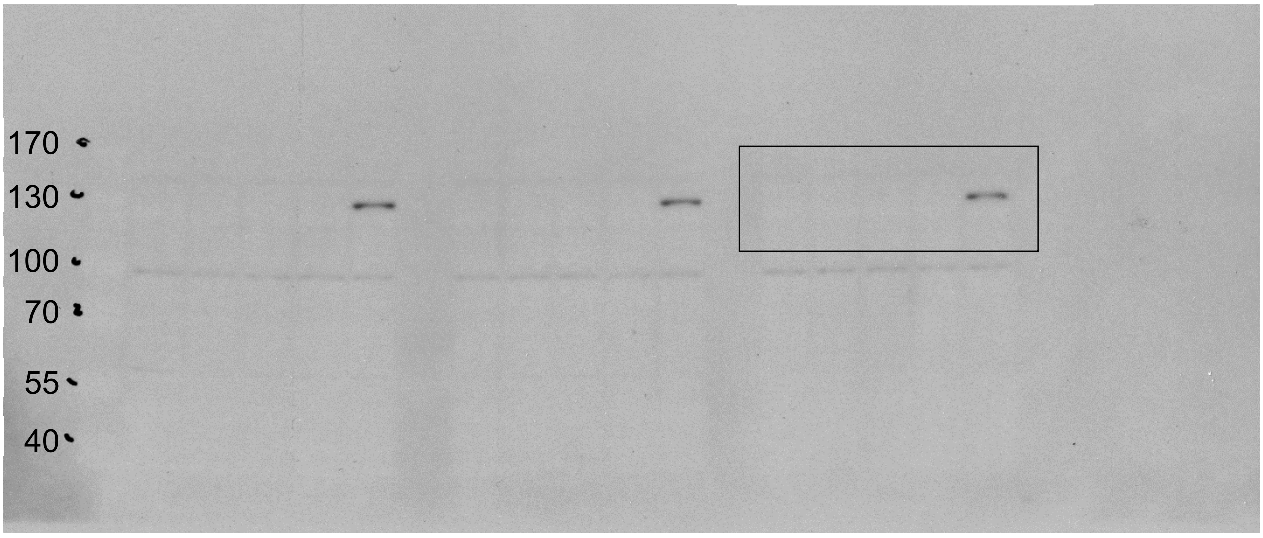

anti-Hsp104

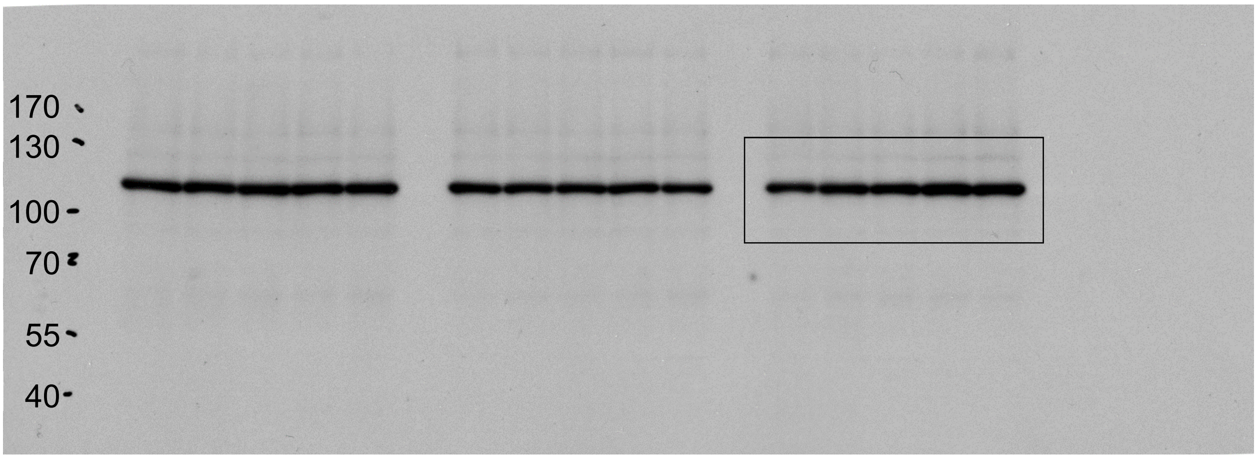

Figure 1

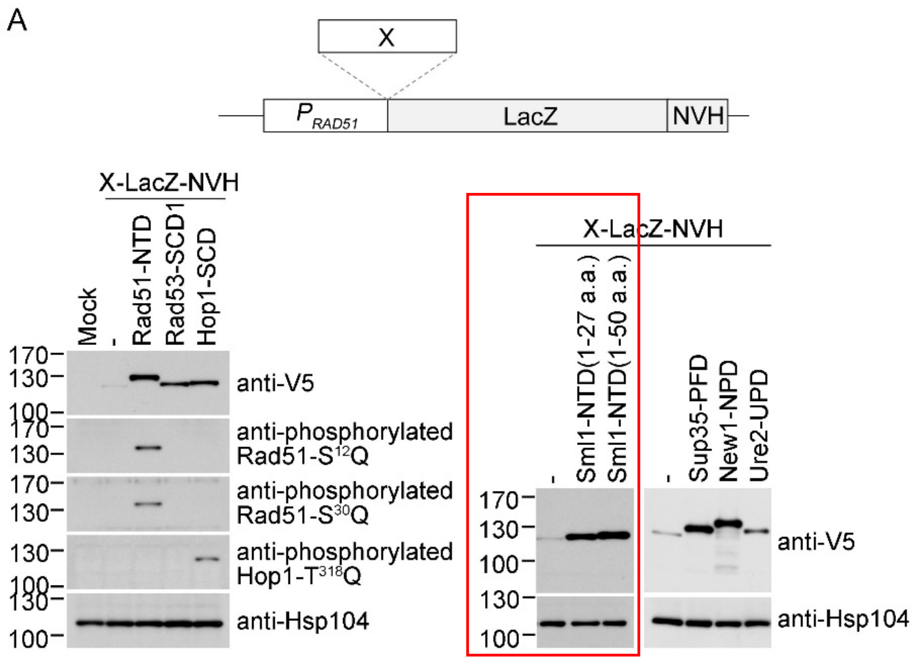

Sml1-NTD

anti-V5

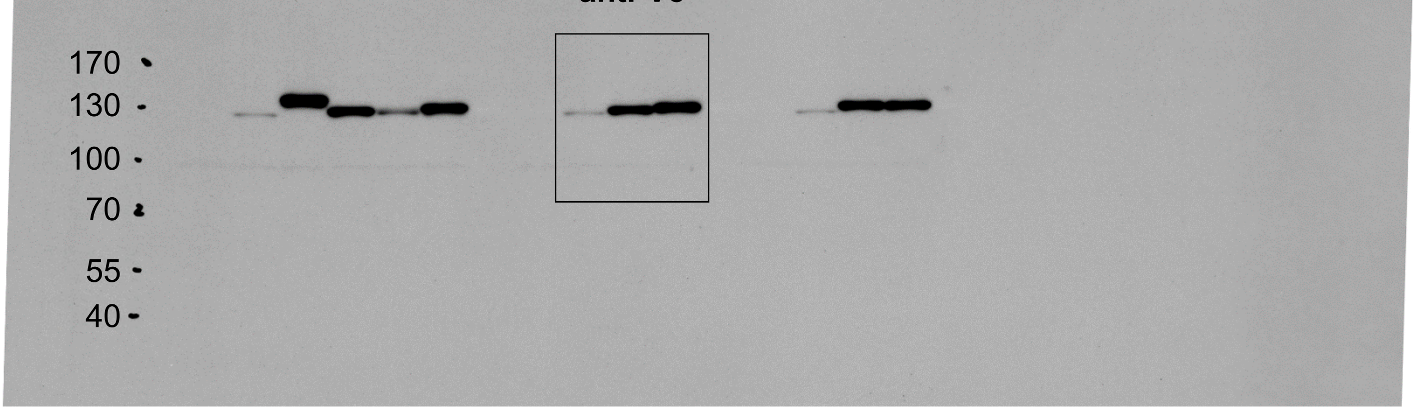

Sml1-NTD

anti-Hsp104

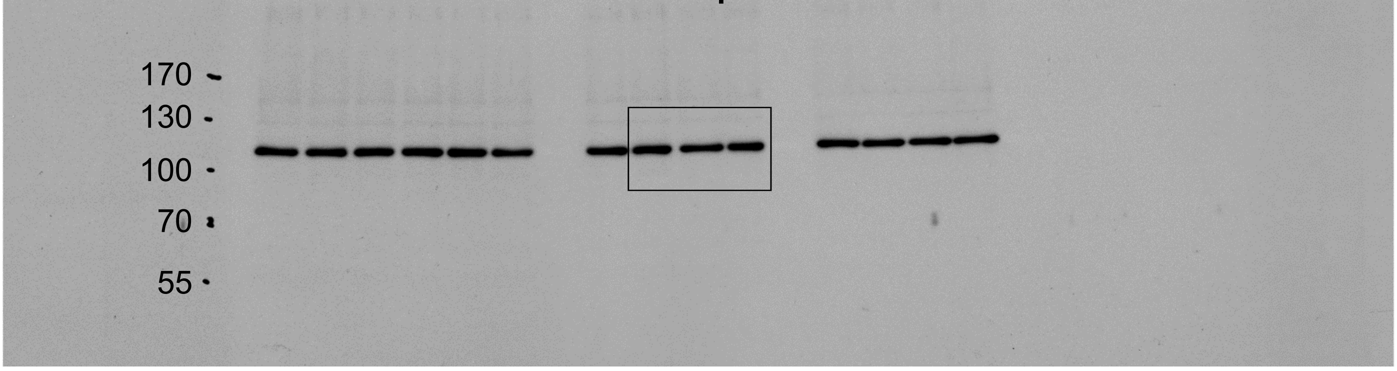

Figure 1

A

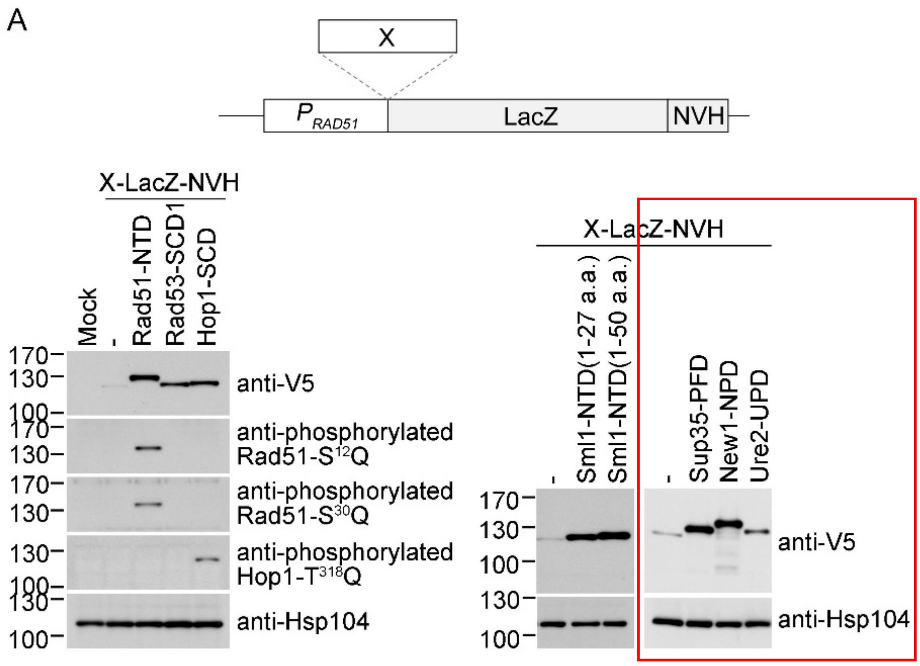

Sup35, New1 and Ure2

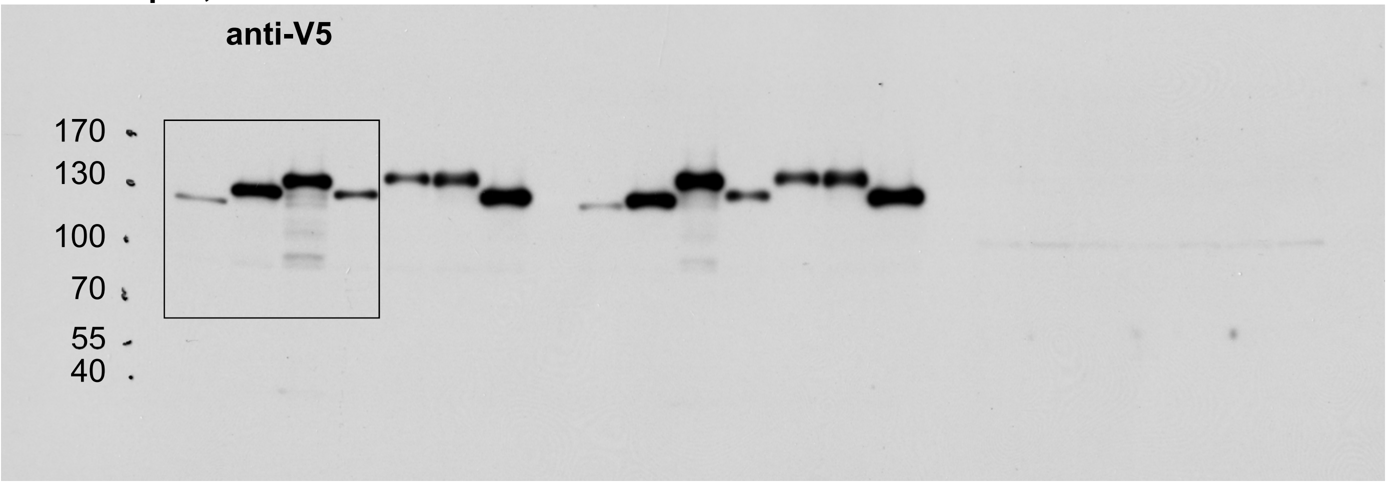

Sup35, New1 and Ure2

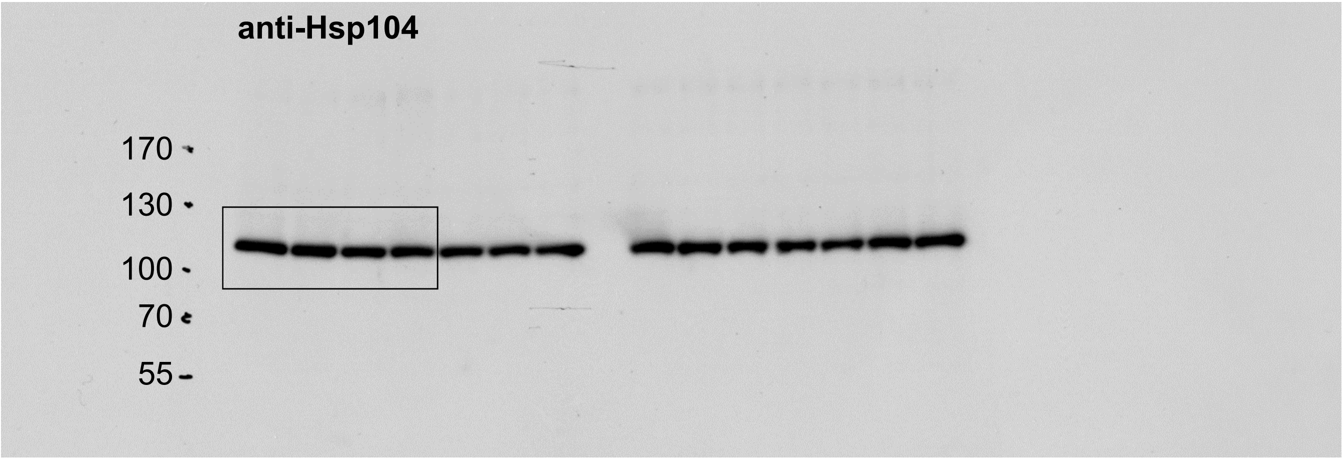

Figure 1

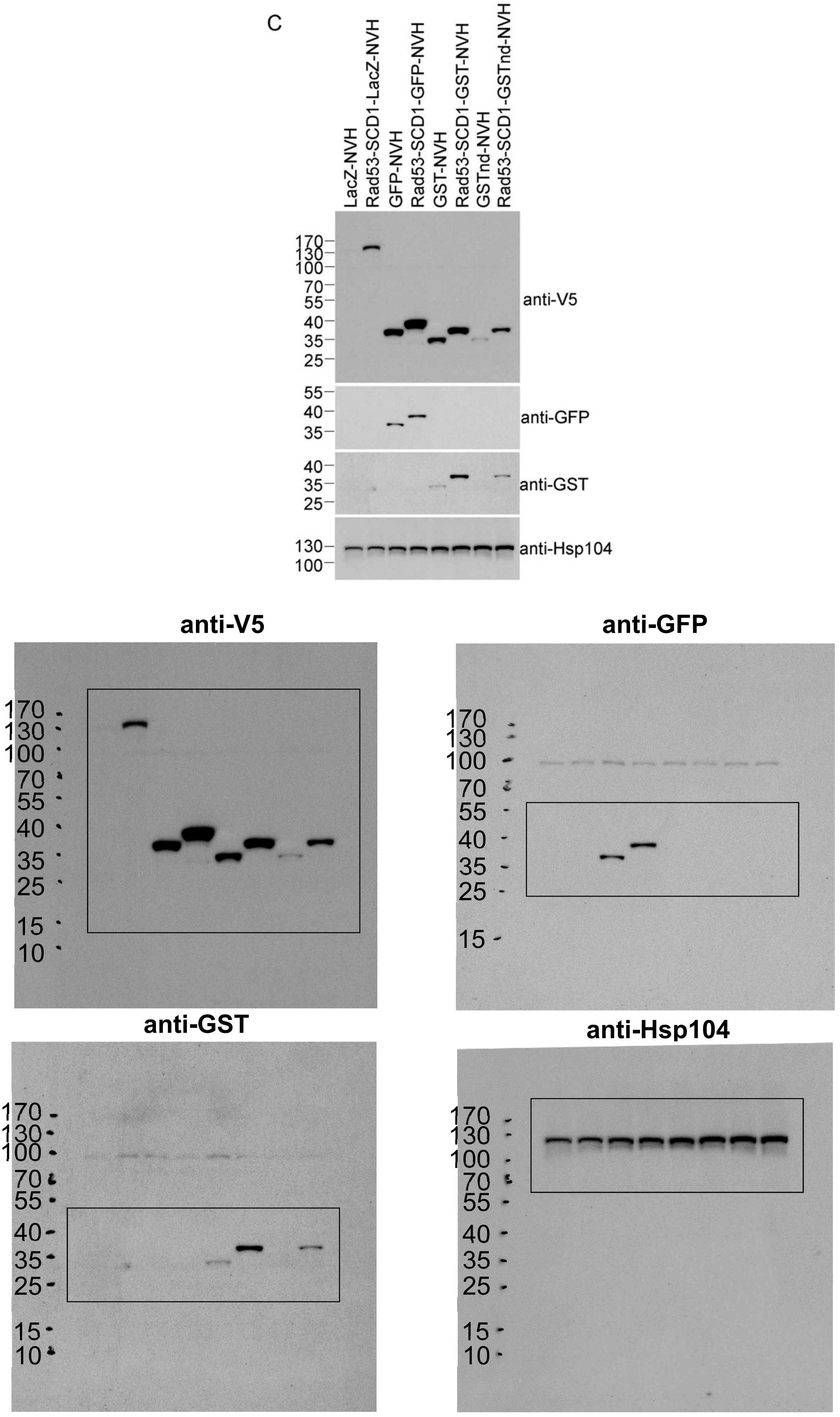
C
LacZ-NVH
Rad53-SCD1-LacZ-NVH
GFP-NVH
Rad53-SCD1-GFP-NVH
GST-NVH
Rad53-SCD1-GST-NVH
GSTnd-NVH
Rad53-SCD1-GSTnd-NVH
170
130
100
70
55
40
35
25
anti-V5
anti-GFP
anti-GST
anti-Hsp104
anti-V5
anti-GFP
anti-GST
anti-Hsp104
15
10

Figure 1

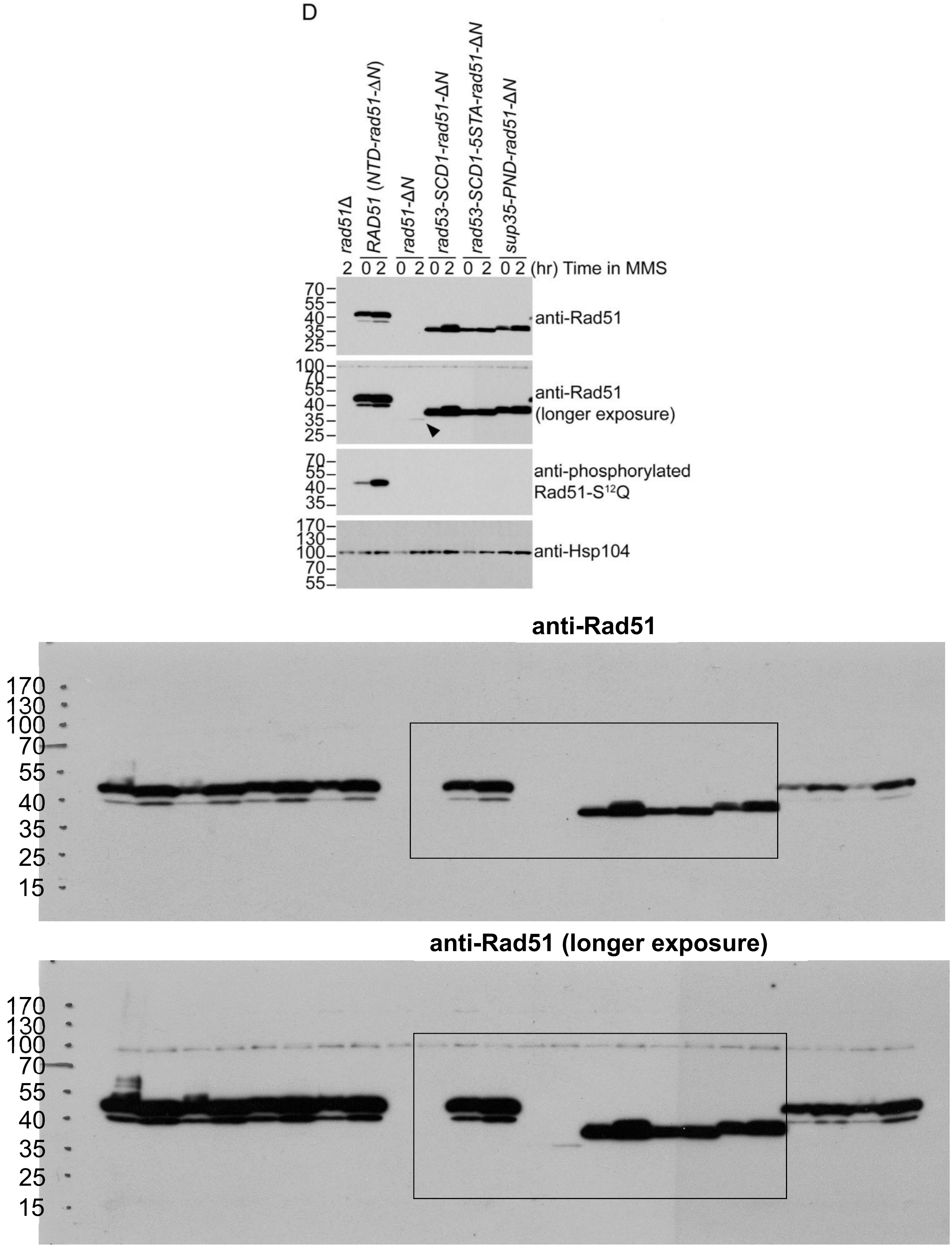
D
rad51Δ
RAD51 (NTD-rad51-ΔN)
rad51-ΔN
rad53-SCD1-rad51-ΔN
rad53-SCD1-5STA-rad51-ΔN
sup35-PND-rad51-ΔN
2 0 2 0 2 0 2 0 2 0 2 (hr) Time in MMS
70
55
40
35
25
anti-Rad51
100
70
55
40
35
25
anti-Rad51 (longer exposure)
70
55
40
35
anti-phosphorylated Rad51-S12Q
170
130
100
70
55
anti-Hsp104
anti-Rad51
170
130
100
70
55
40
35
25
15
anti-Rad51 (longer exposure)
170
130
100
70
55
40
35
25
15

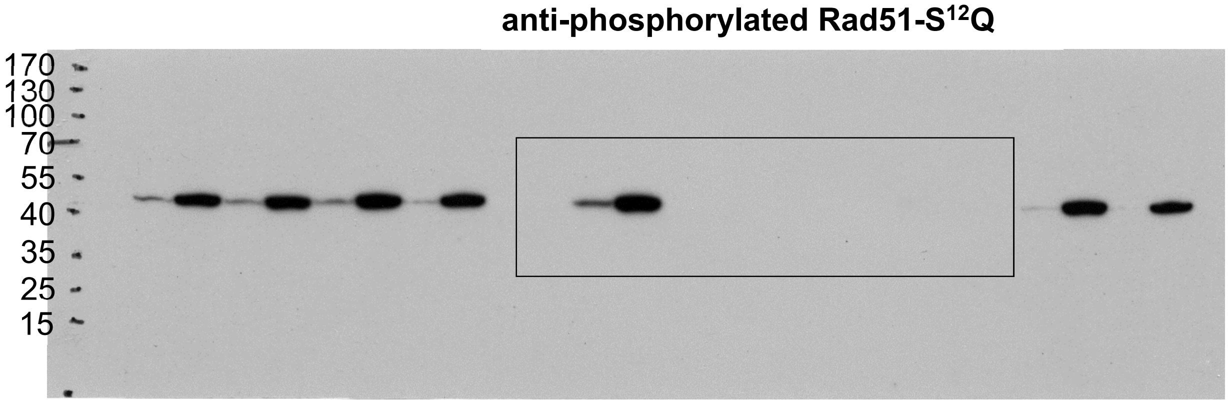
anti-phosphorylated Rad51-S12Q
170
130
100
70
55
40
35
25
15

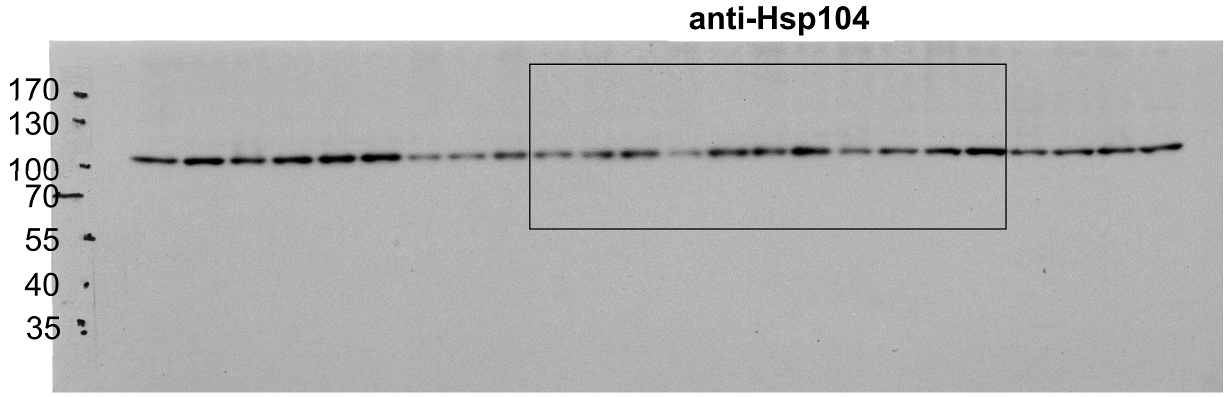
anti-Hsp104
170
130
100
70
55
40
35
