# Supplementary figures and images for: Noncanonical usage of stop codons in ciliates expands proteins with structurally flexible Q-rich motifs
Source: eLife. 2024 Feb 23;12:RP91405. doi: 10.7554/eLife.91405 (PMC10942620; doi:10.7554/eLife.91405)

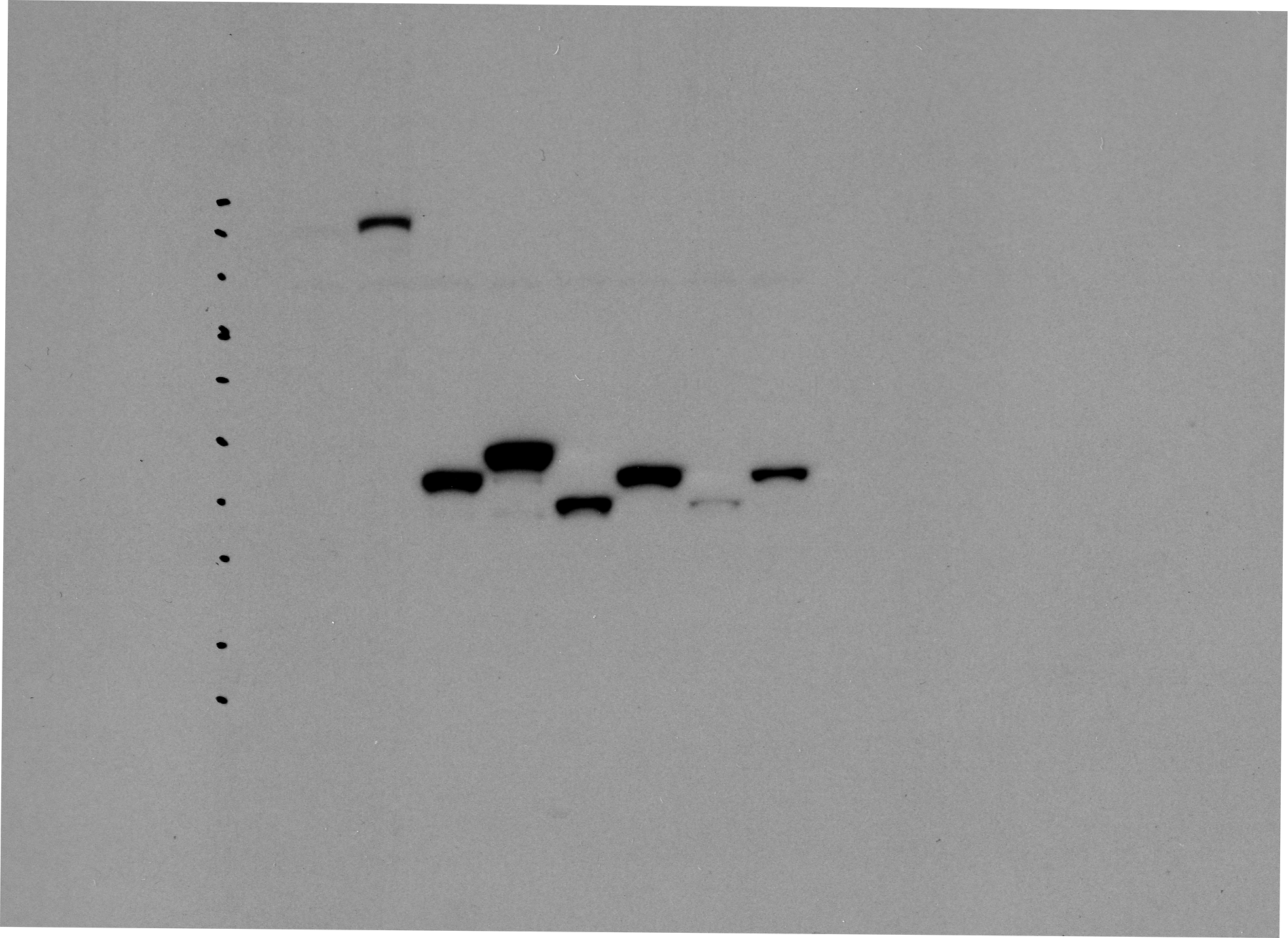

Supplement: Figure 1—source data 1. [file elife-91405-fig1-data1.zip › Figure1/C/anti-V5.tif]

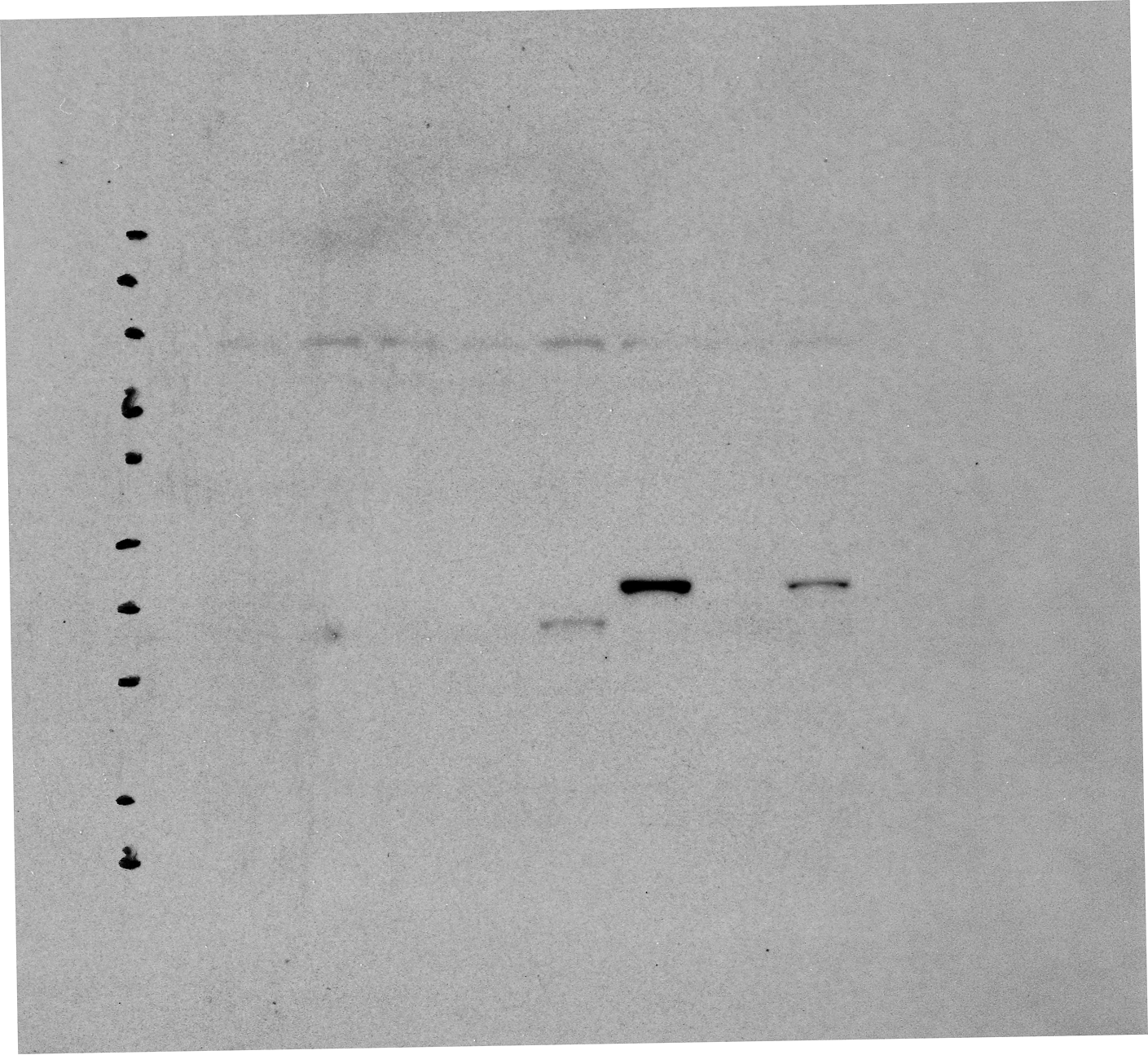

Supplement: Figure 1—source data 1. [file elife-91405-fig1-data1.zip › Figure1/C/anti-GST.tif]

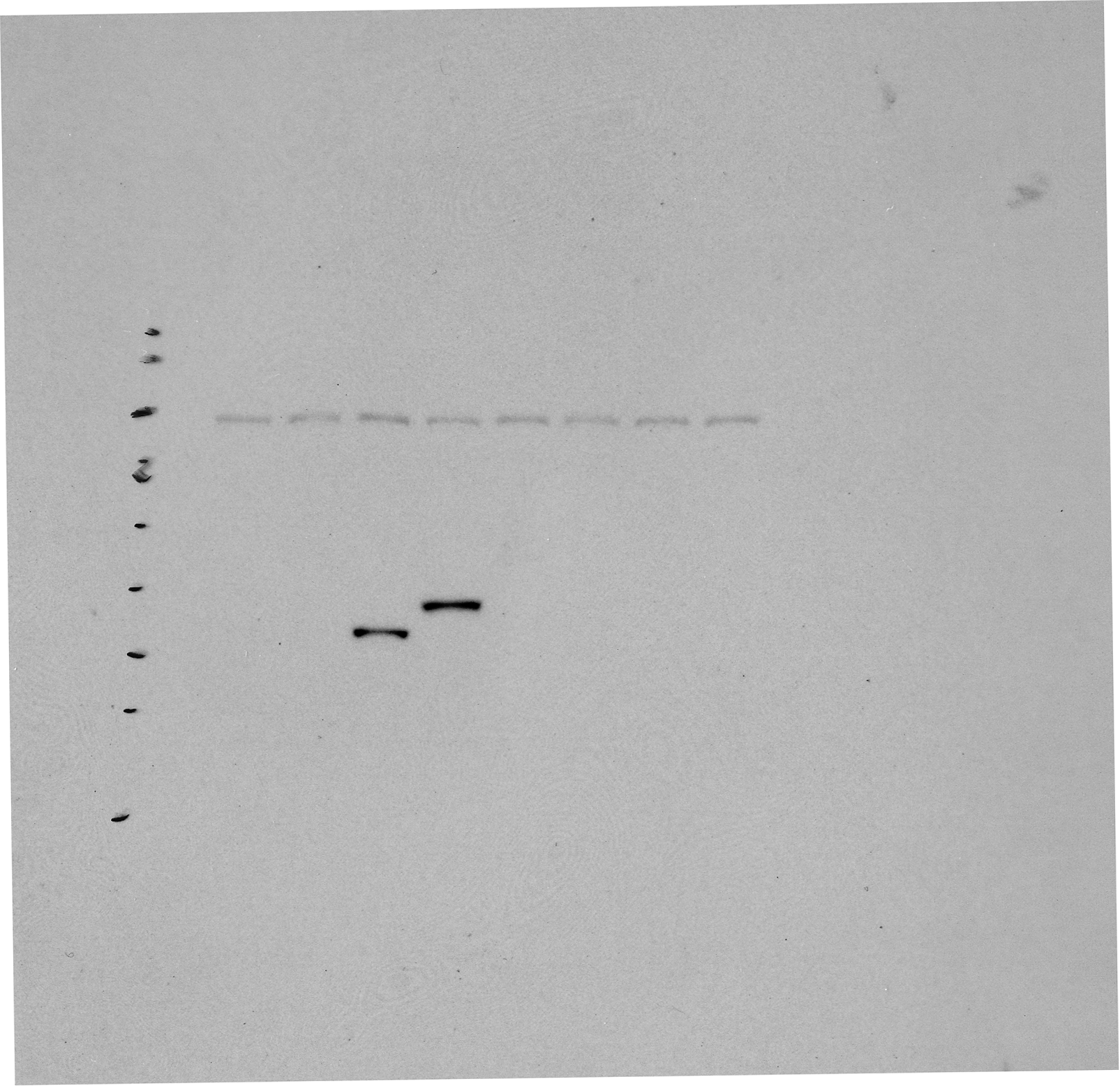

Supplement: Figure 1—source data 1. [file elife-91405-fig1-data1.zip › Figure1/C/anti-GFP.tif]

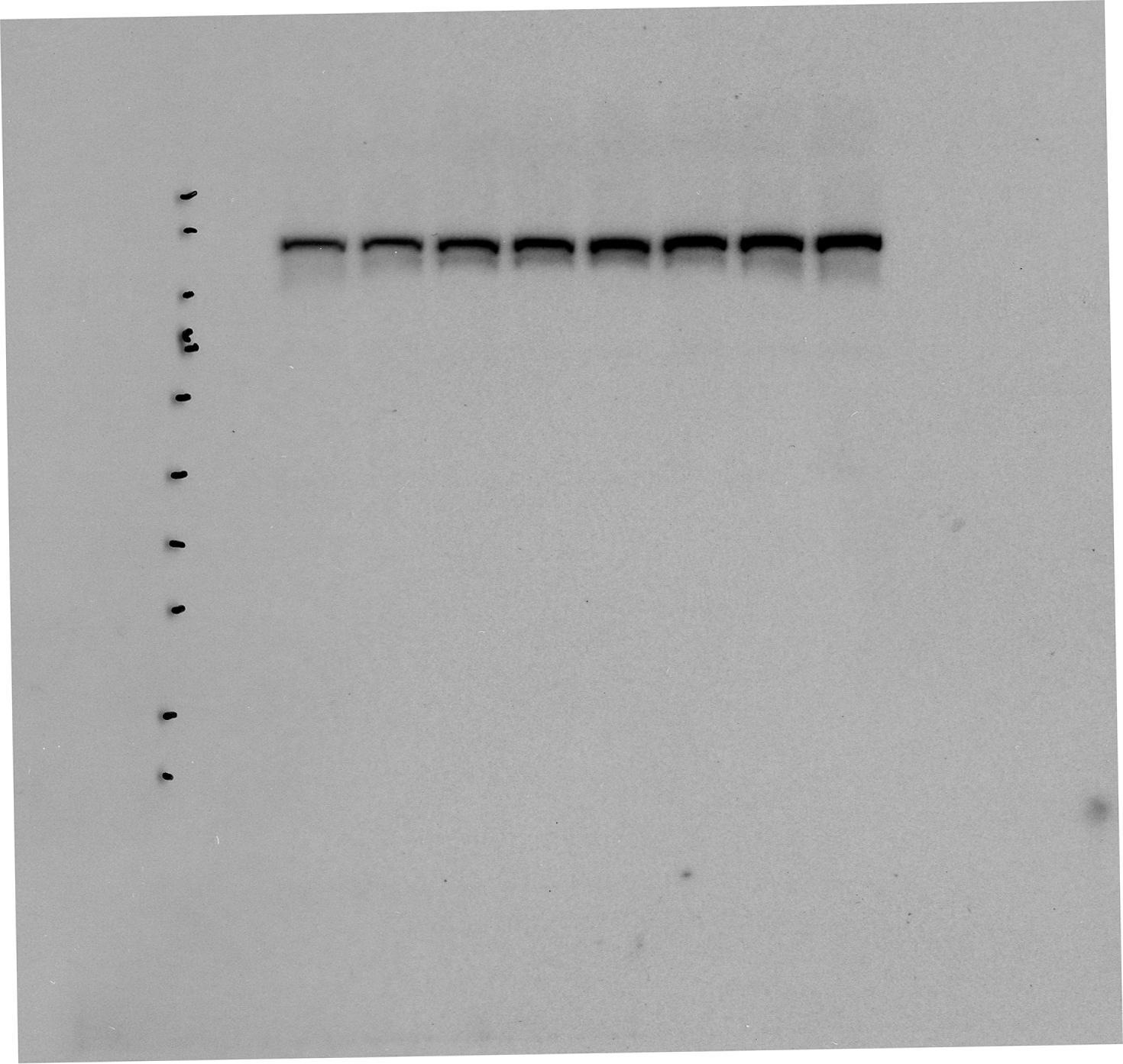

Supplement: Figure 1—source data 1. [file elife-91405-fig1-data1.zip › Figure1/C/anti-Hsp.tif]

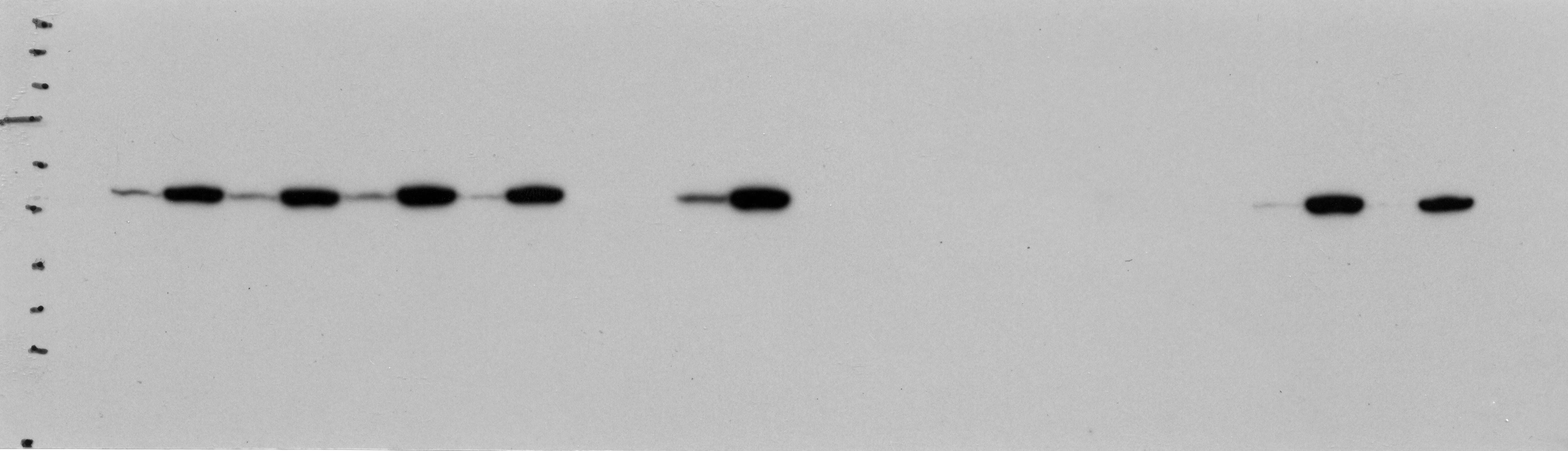

Supplement: Figure 1—source data 1. [file elife-91405-fig1-data1.zip › Figure1/D/anti-Rad51-P12 3min044.tif]

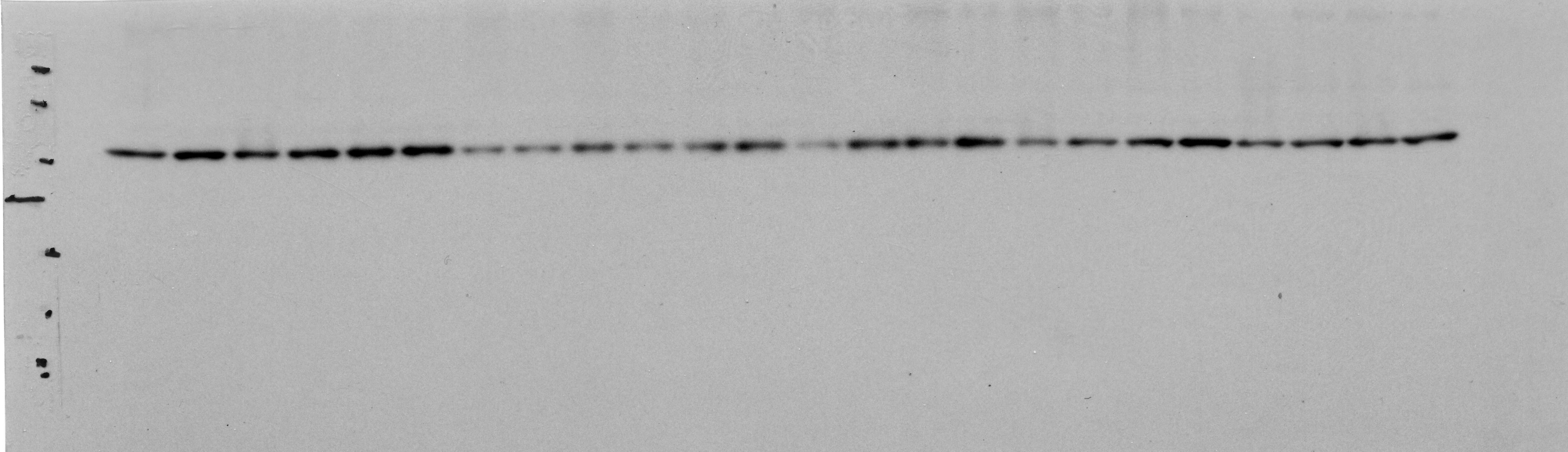

Supplement: Figure 1—source data 1. [file elife-91405-fig1-data1.zip › Figure1/D/anti-Hsp104 10sec045.tif]

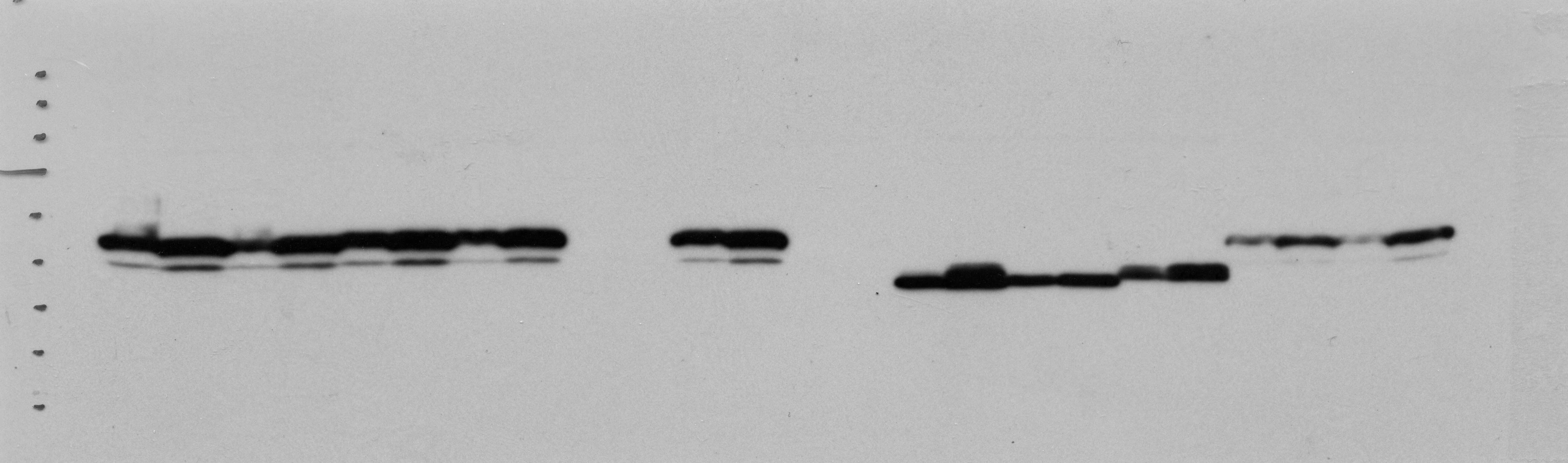

Supplement: Figure 1—source data 1. [file elife-91405-fig1-data1.zip › Figure1/D/anti-Rad51 1min039.tif]

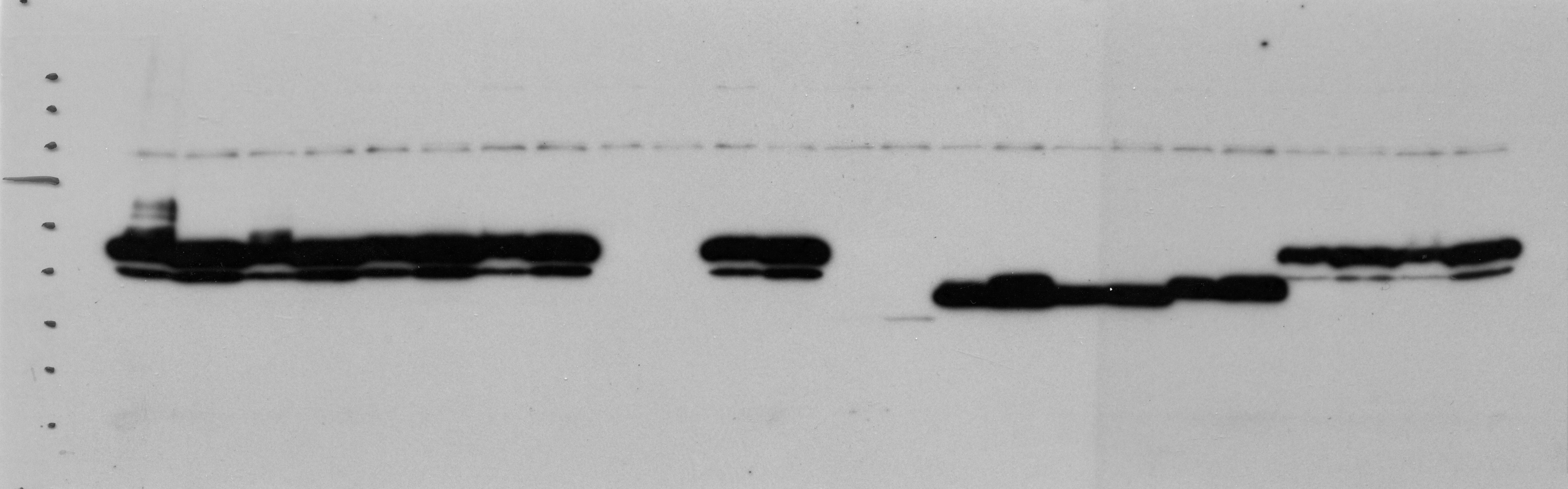

Supplement: Figure 1—source data 1. [file elife-91405-fig1-data1.zip › Figure1/D/anti-Rad51 10min040.tif]

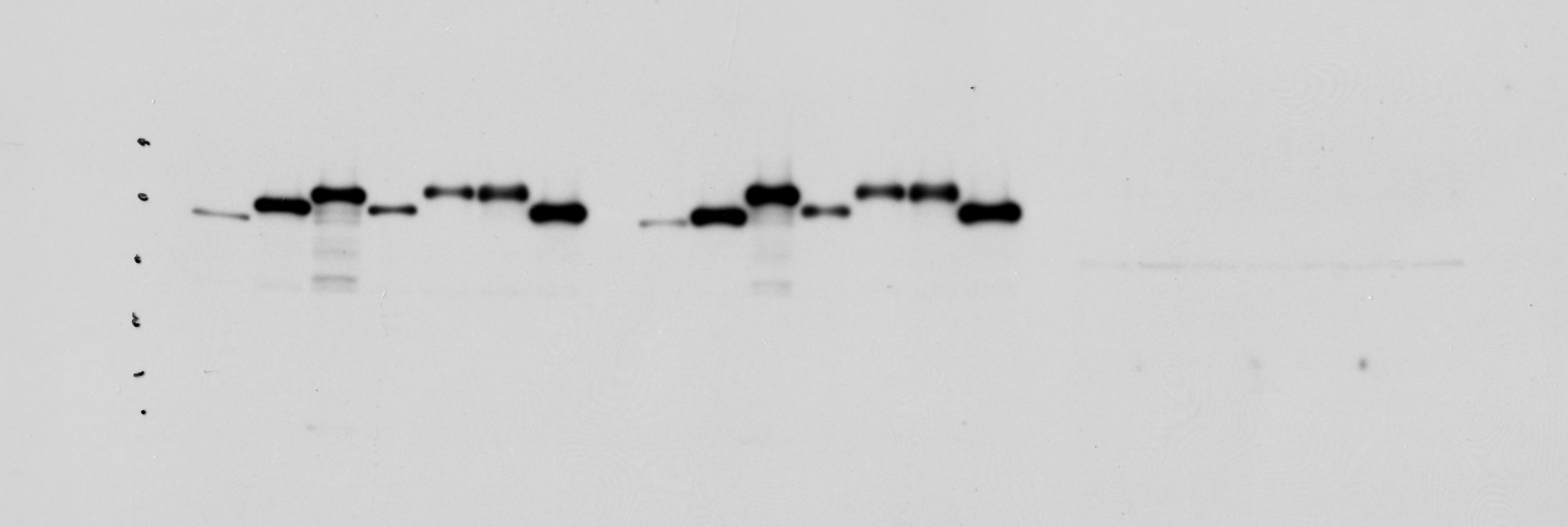

Supplement: Figure 1—source data 1. [file elife-91405-fig1-data1.zip › Figure1/A/right/Sup35 New1 Ure2 anti-V5 .tif]

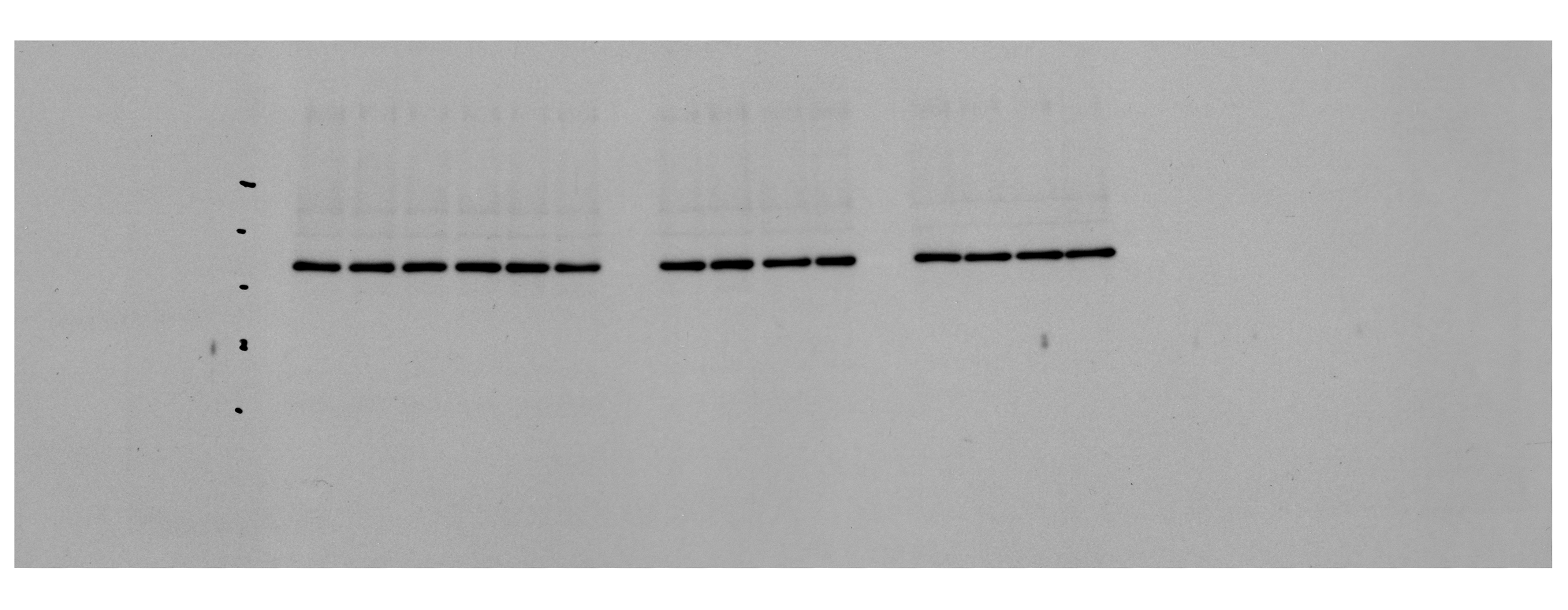

Supplement: Figure 1—source data 1. [file elife-91405-fig1-data1.zip › Figure1/A/right/Sml1-anti-Hsp104.tif]

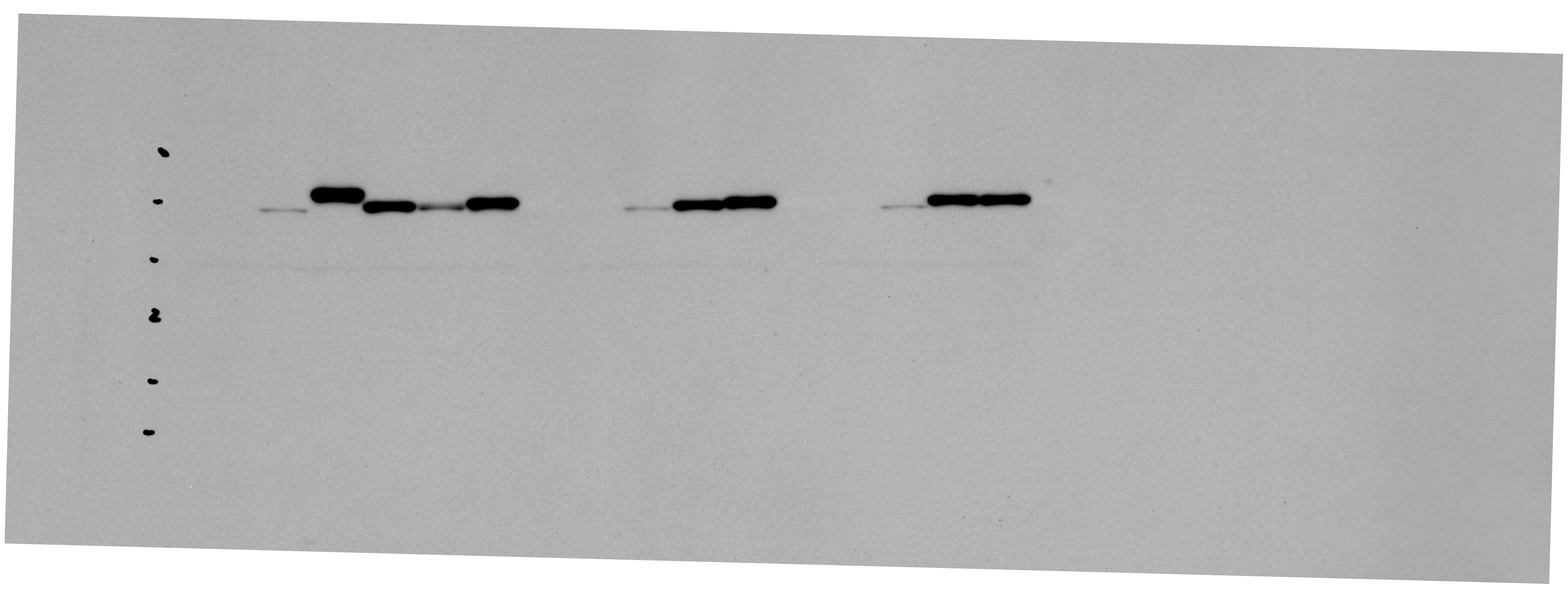

Supplement: Figure 1—source data 1. [file elife-91405-fig1-data1.zip › Figure1/A/right/Sml1-anti-V5.tif]

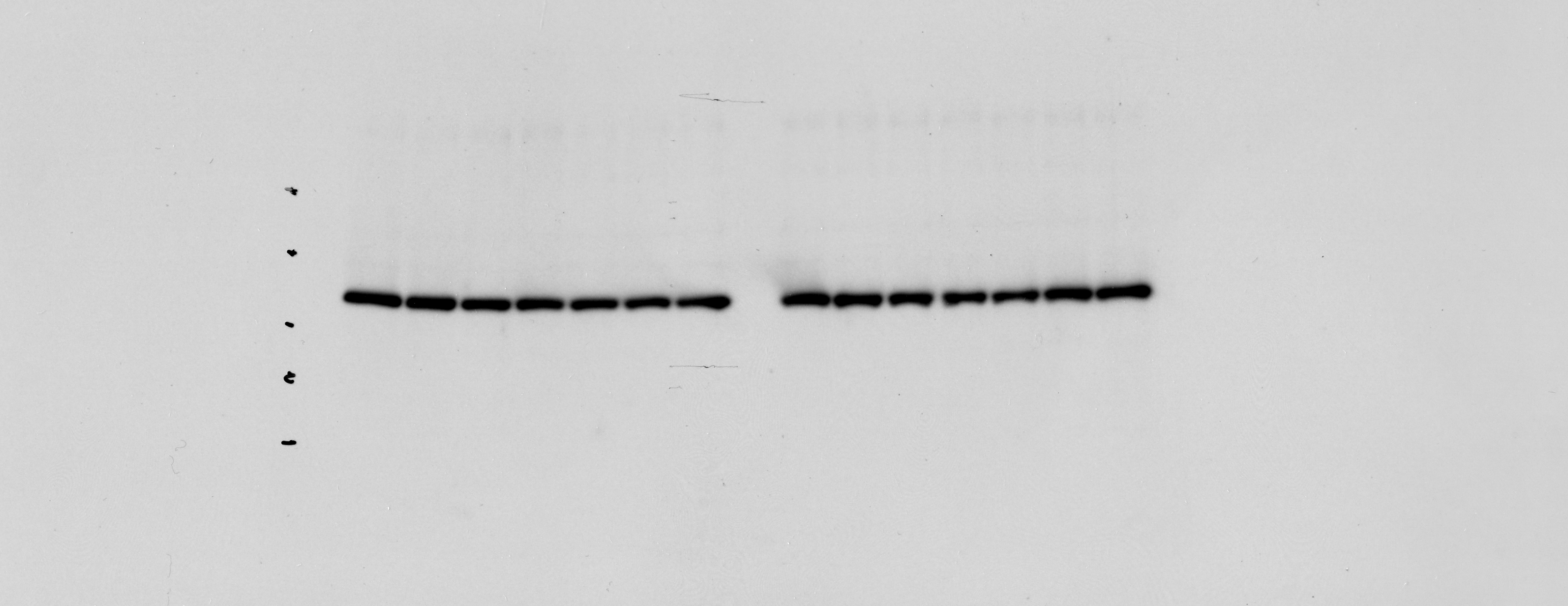

Supplement: Figure 1—source data 1. [file elife-91405-fig1-data1.zip › Figure1/A/right/Sup35 New1 Ure2 anti-hsp104 .tif]

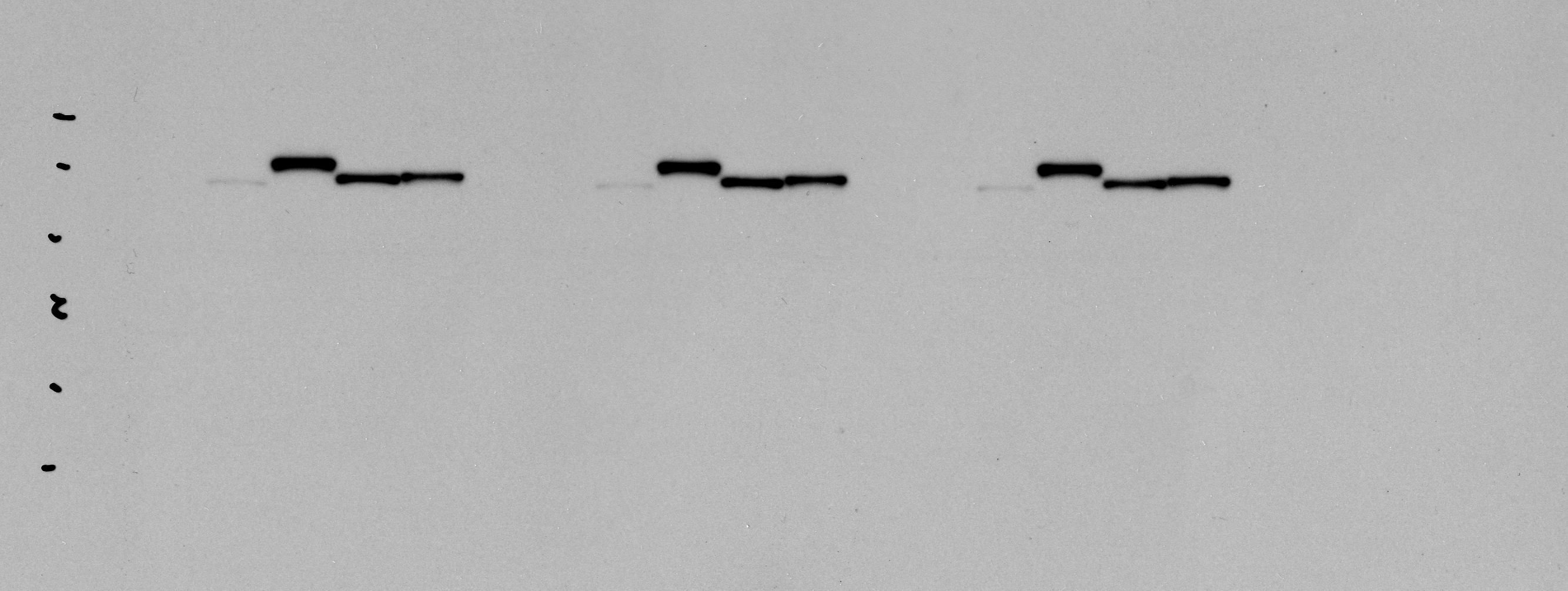

Supplement: Figure 1—source data 1. [file elife-91405-fig1-data1.zip › Figure1/A/left/anti-V5 30sec008.tif]

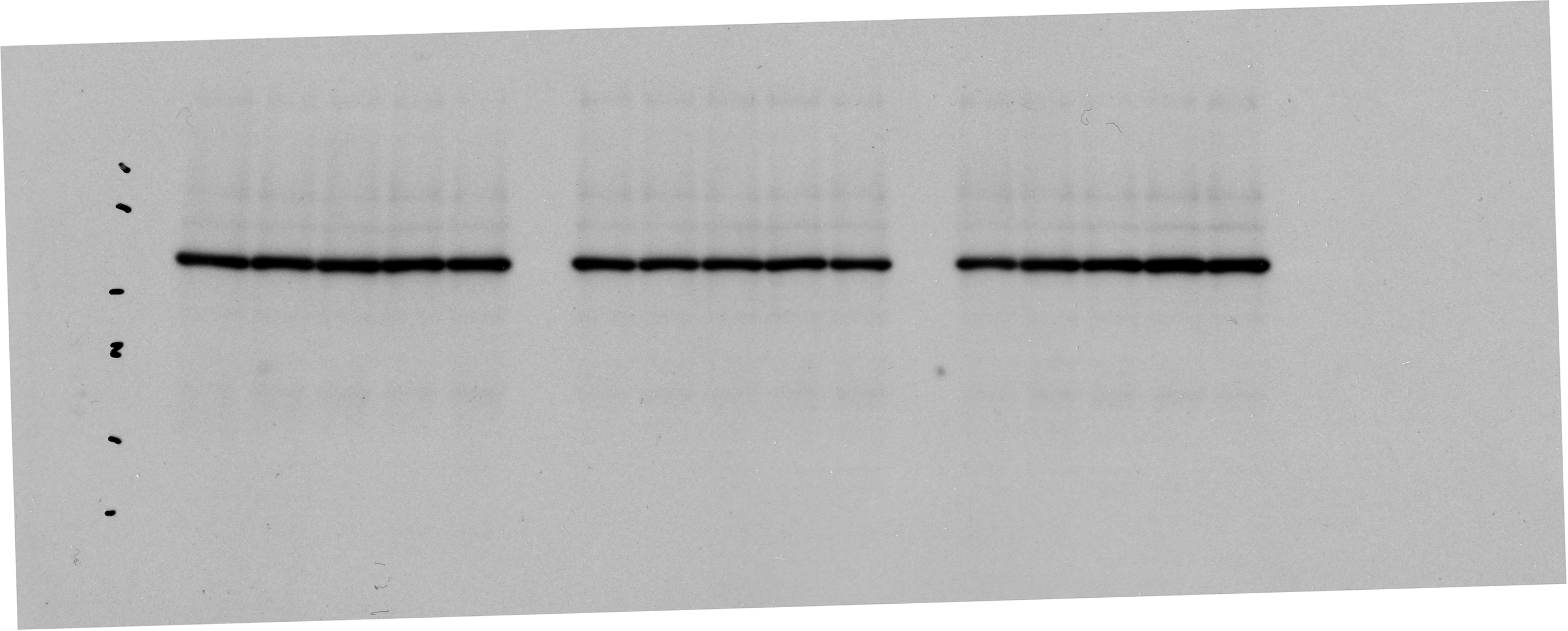

Supplement: Figure 1—source data 1. [file elife-91405-fig1-data1.zip › Figure1/A/left/anti-Hsp104 5sec011.tif]

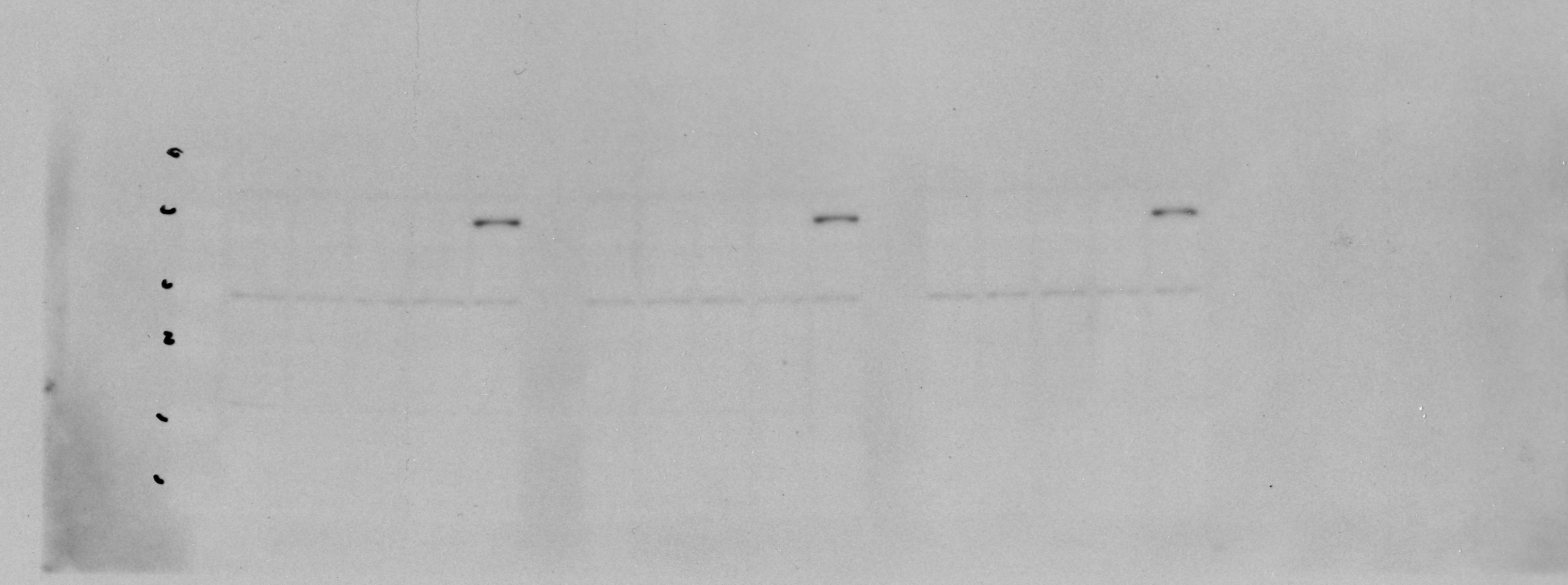

Supplement: Figure 1—source data 1. [file elife-91405-fig1-data1.zip › Figure1/A/left/anti-Hop1-T318 2min012.tif]

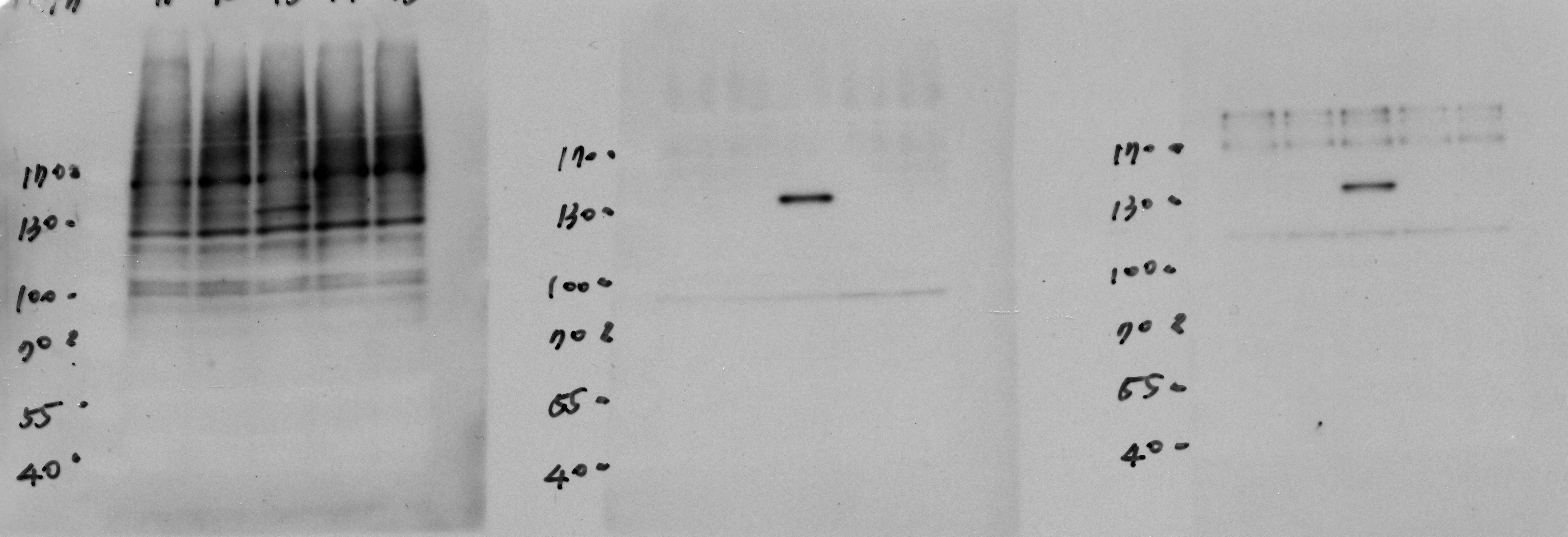

Supplement: Figure 1—source data 1. [file elife-91405-fig1-data1.zip › Figure1/A/left/anti-p-Rad51 1min047.tif]

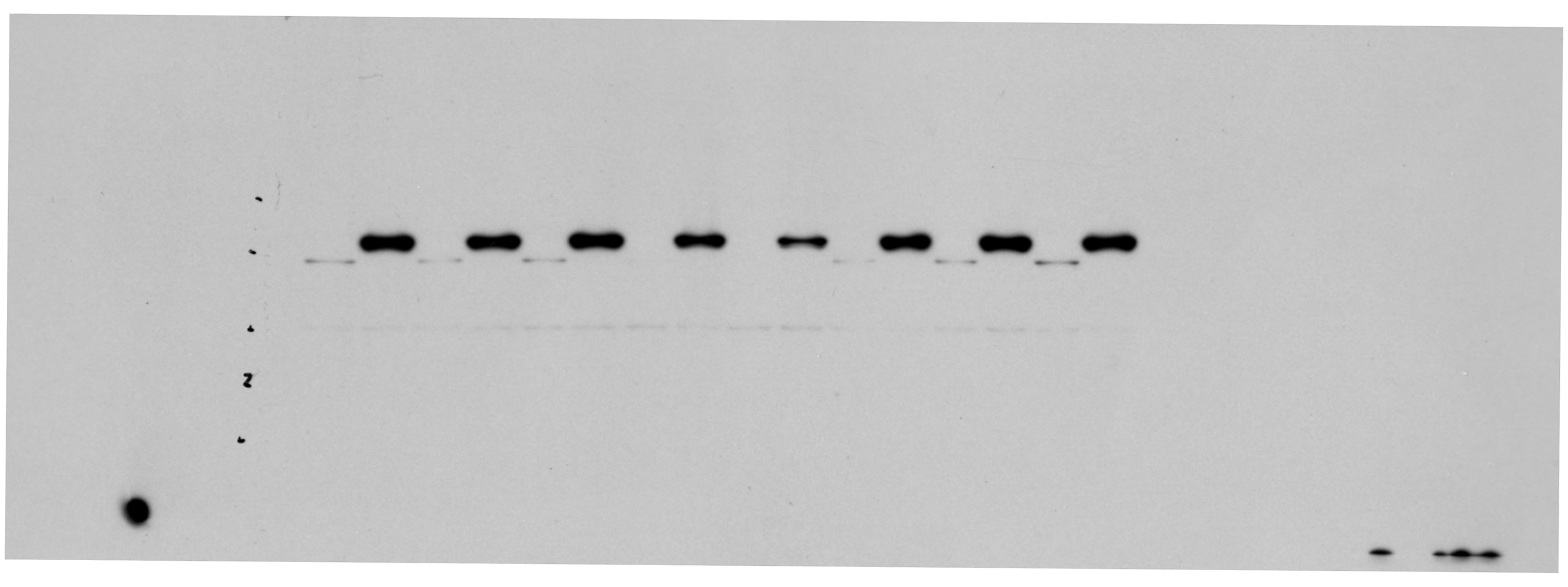

Supplement: Figure 3—source data 1. [file elife-91405-fig3-data1.zip › Figure3/anti-V5.tif]

Figure 3

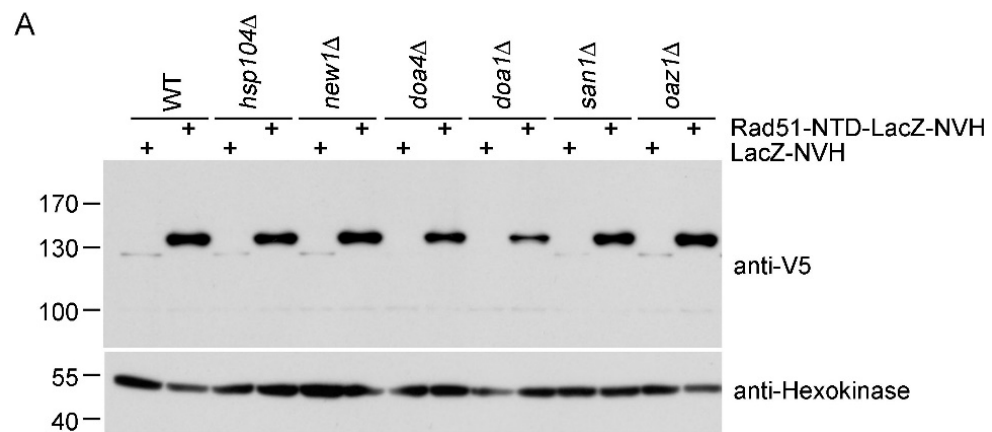

anti-V5

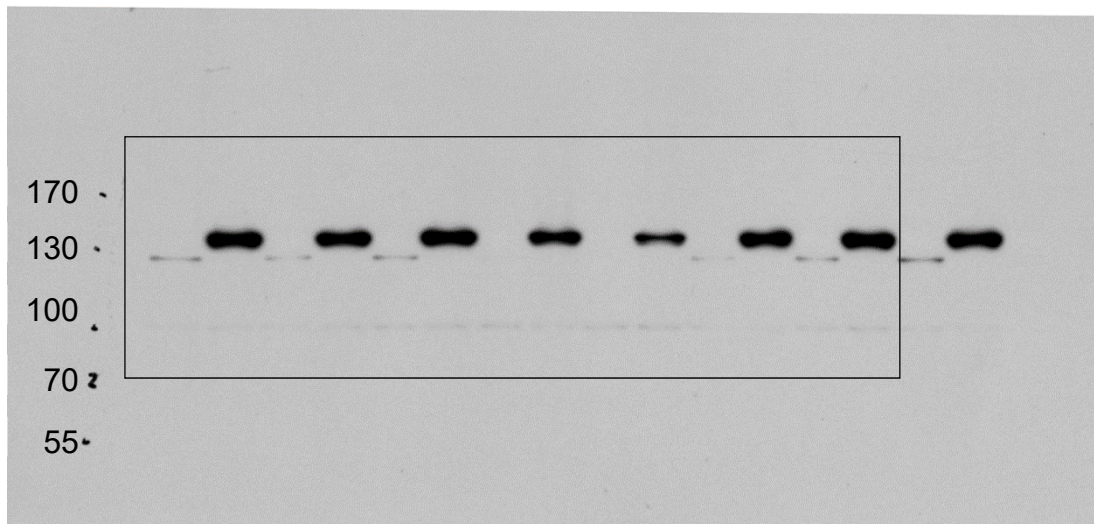

anti-Hexokinase

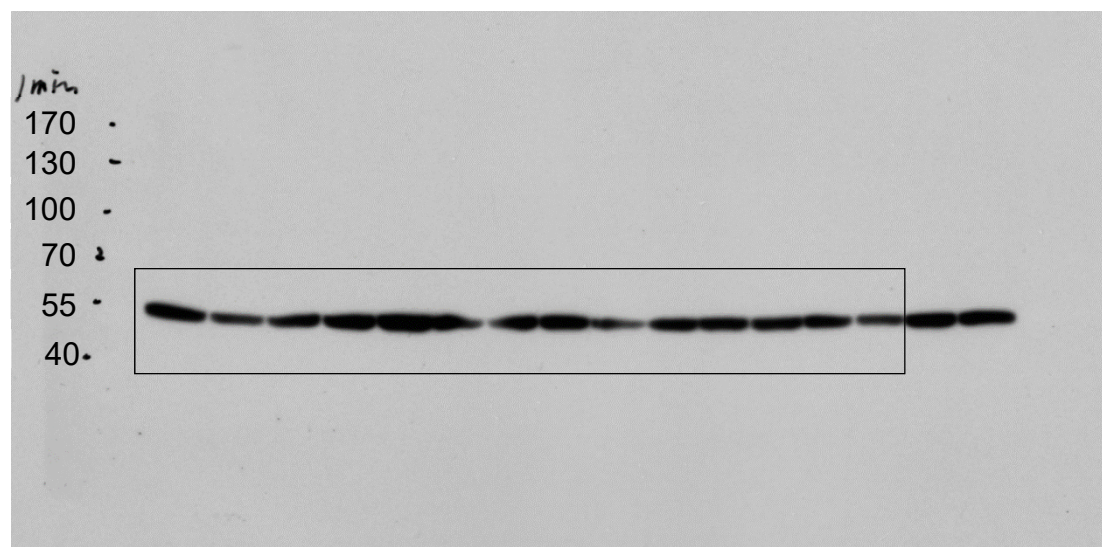

Supplement: Figure 3—source data 1. [file elife-91405-fig3-data1.zip › Figure3/Figure3_source_data_labelled.pdf]

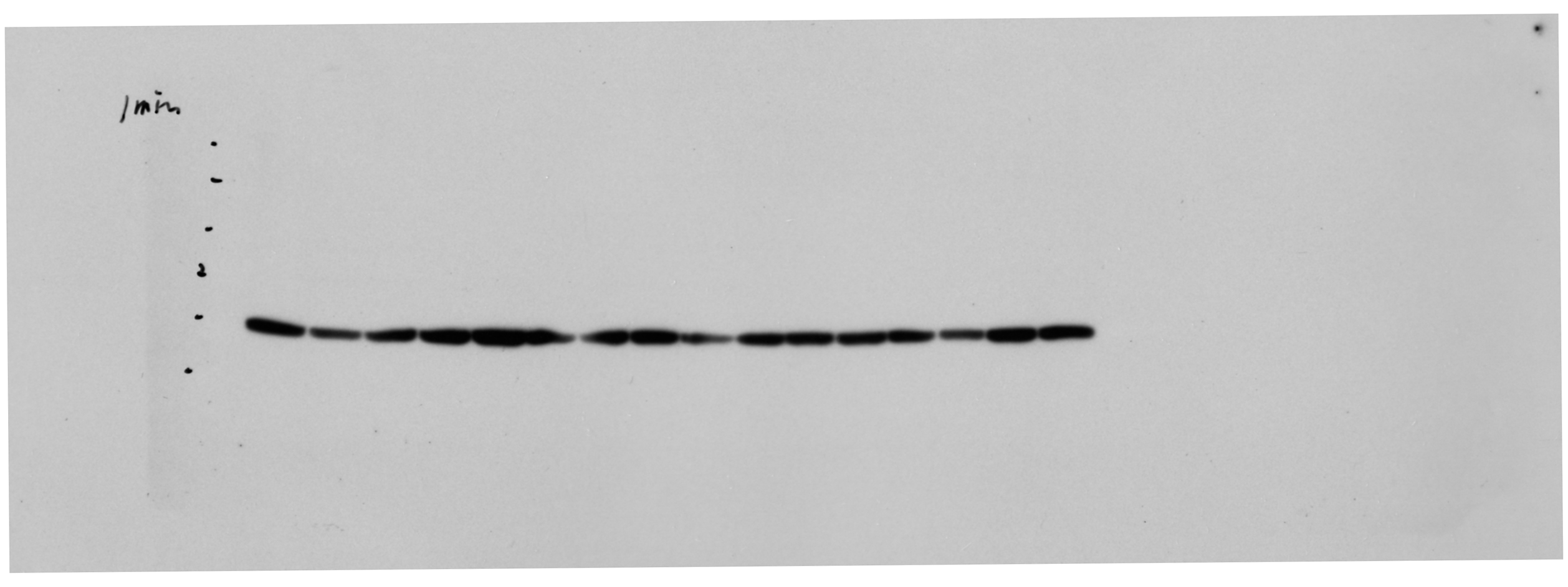

Supplement: Figure 3—source data 1. [file elife-91405-fig3-data1.zip › Figure3/anti-Hexokinase.tif]

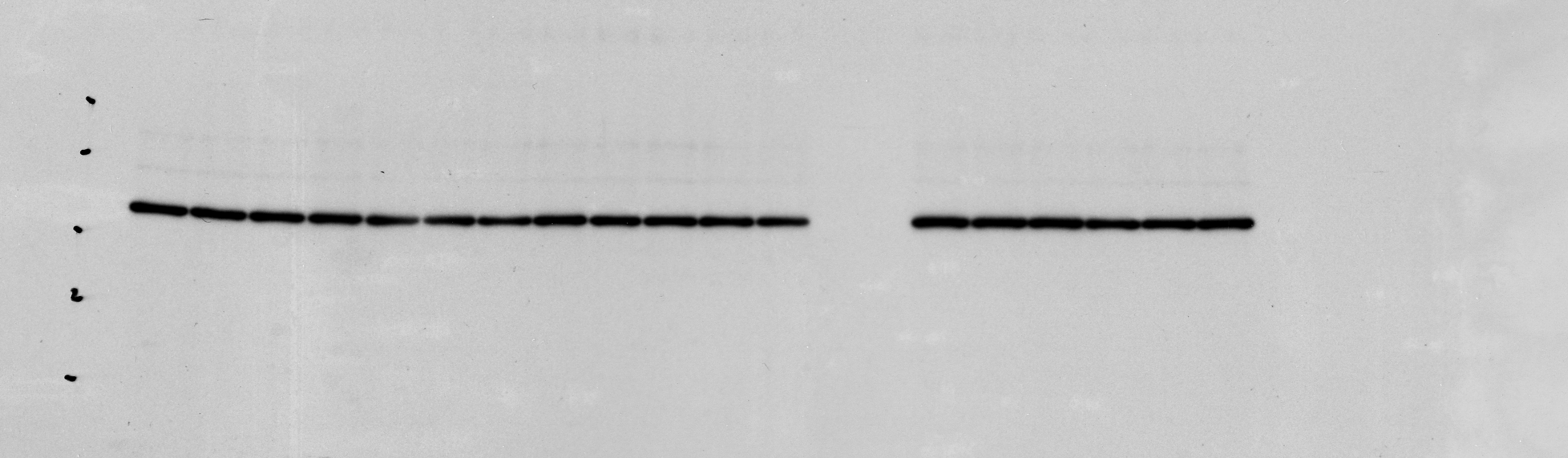

Supplement: Figure 5—source data 1. [file elife-91405-fig5-data1.zip › Figure5/A/anti-Hsp104.tif]

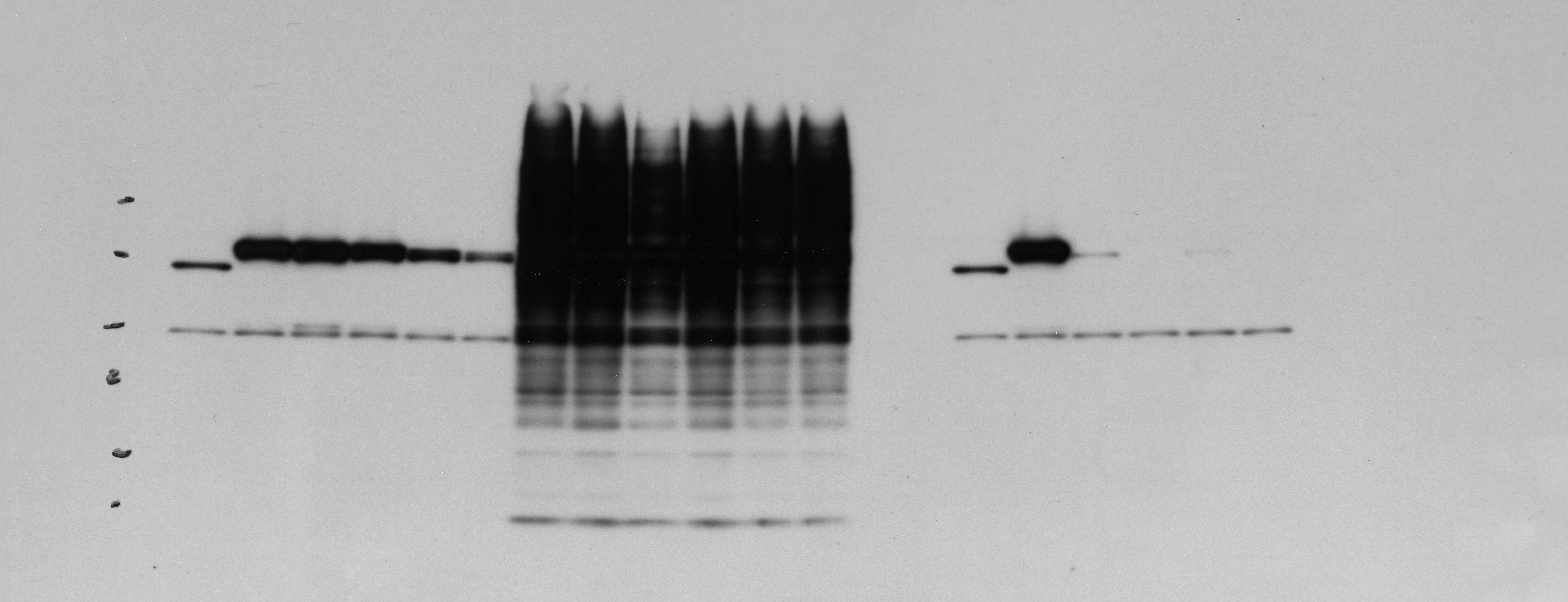

Supplement: Figure 5—source data 1. [file elife-91405-fig5-data1.zip › Figure5/A/anti-V5.tif]

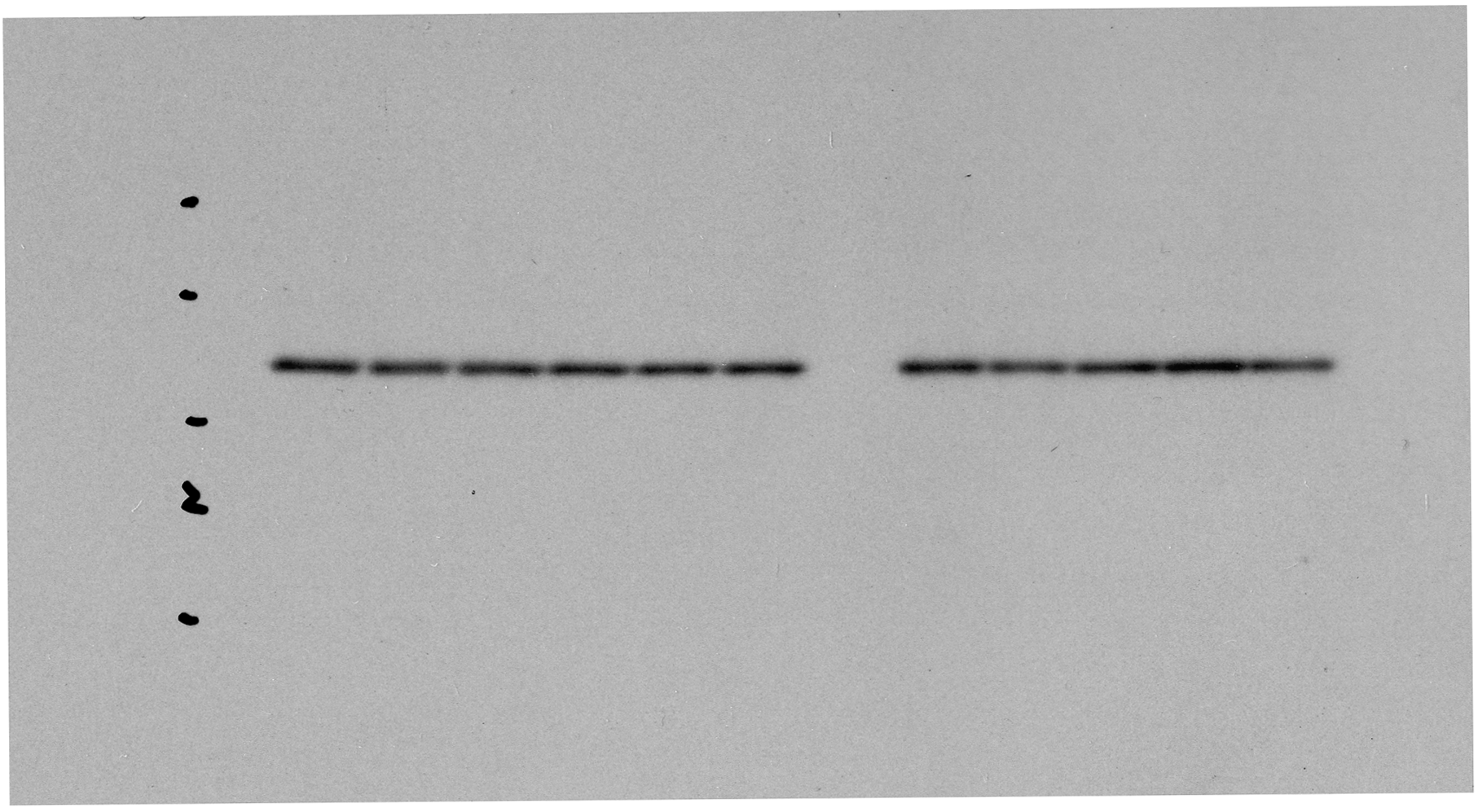

Supplement: Figure 5—source data 1. [file elife-91405-fig5-data1.zip › Figure5/C/anti-Hsp104.tif]

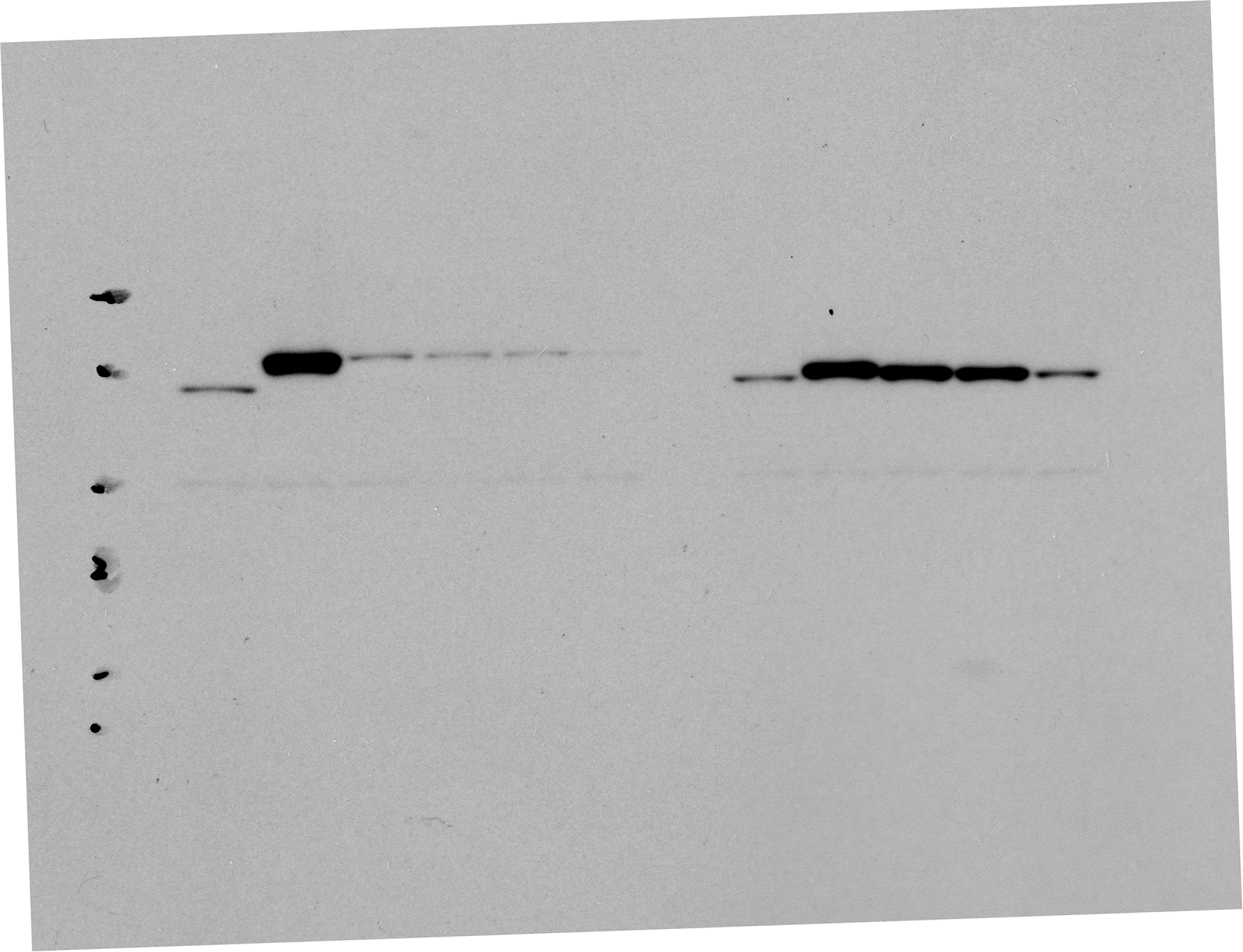

Supplement: Figure 5—source data 1. [file elife-91405-fig5-data1.zip › Figure5/C/anti-V5.tif]

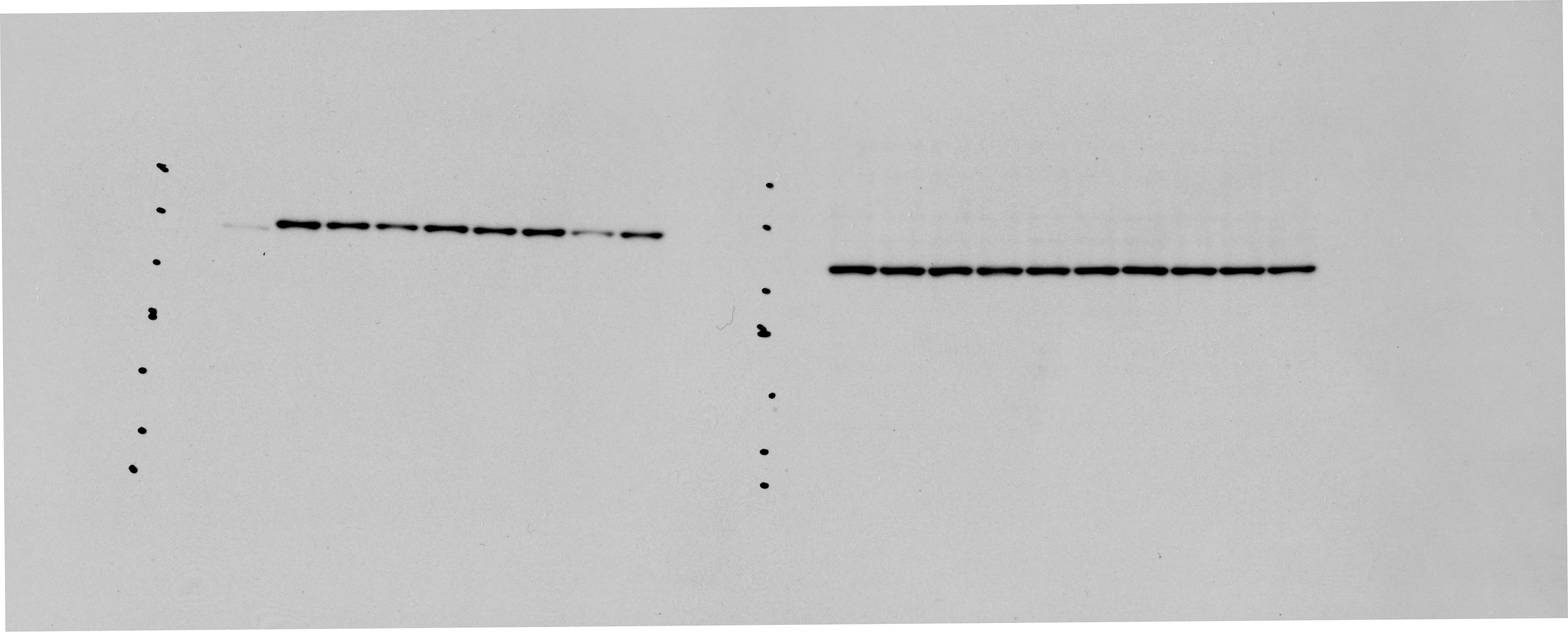

Supplement: Figure 5—source data 1. [file elife-91405-fig5-data1.zip › Figure5/B/anti-V5-Hsp104.tif]

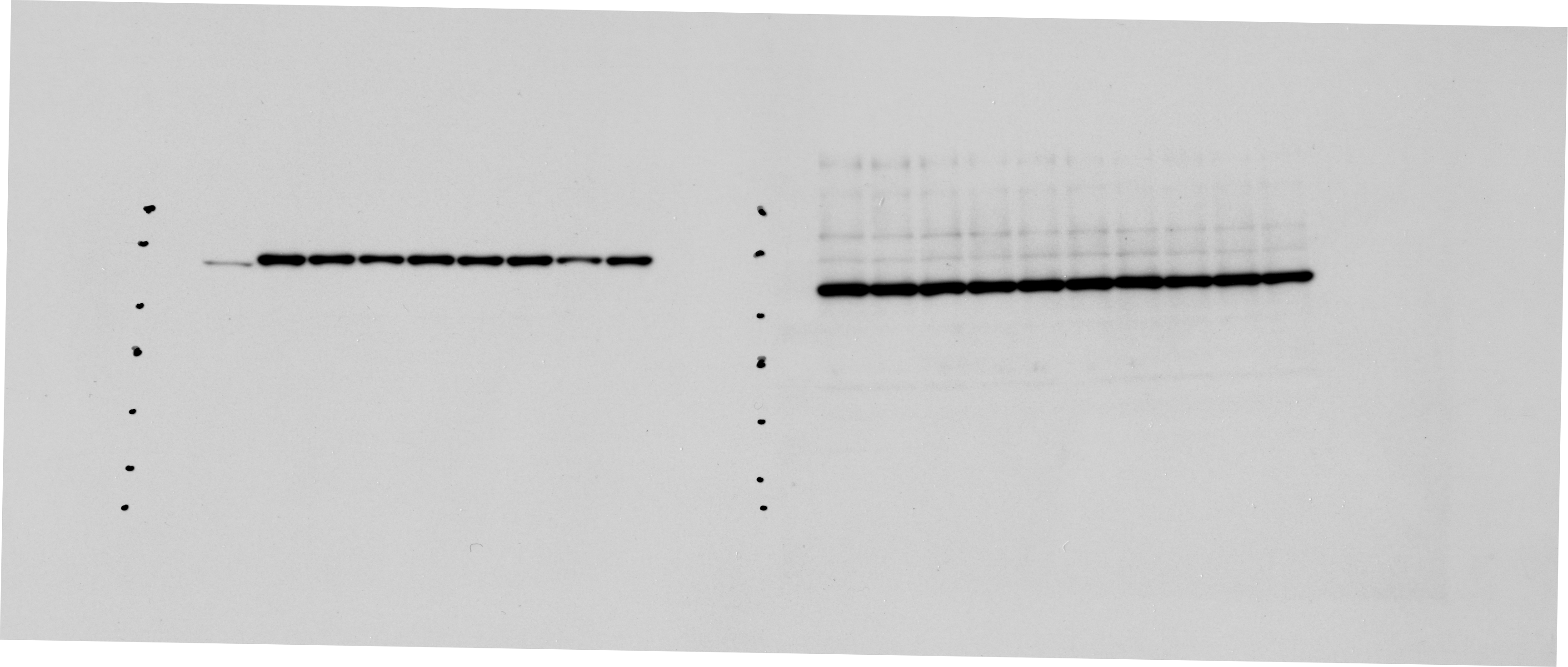

Supplement: Figure 5—source data 1. [file elife-91405-fig5-data1.zip › Figure5/B/anti-V5-Hsp104-2.tif]
