# Supplementary material for: Noncanonical usage of stop codons in ciliates expands proteins with structurally flexible Q-rich motifs
Source: eLife. 2024 Feb 23;12:RP91405. doi: 10.7554/eLife.91405 (PMC10942620; doi:10.7554/eLife.91405)

Figure 5

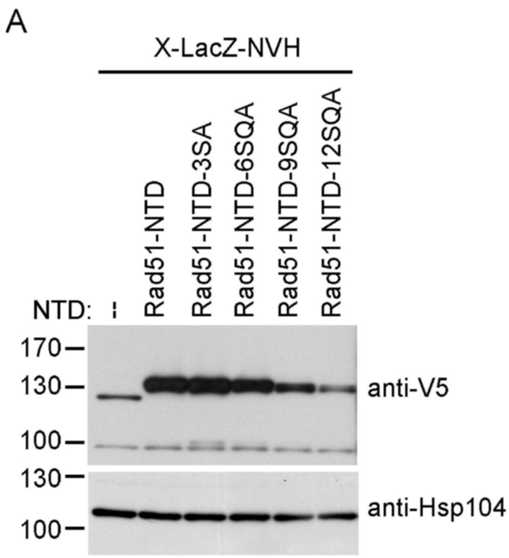

anti-V5

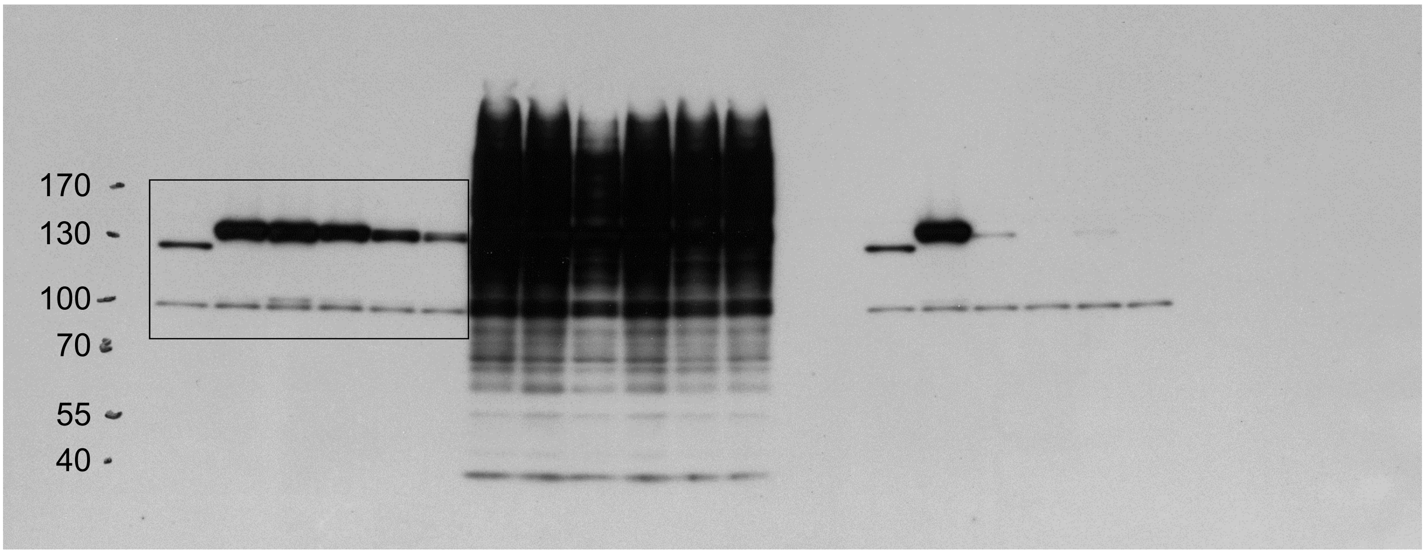

anti-Hsp104

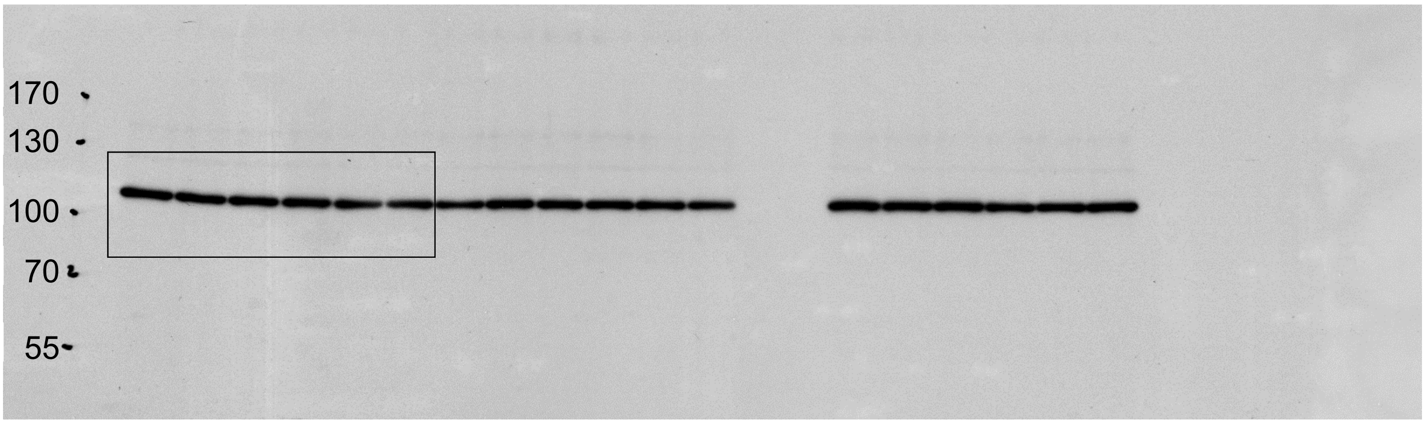

Figure 5

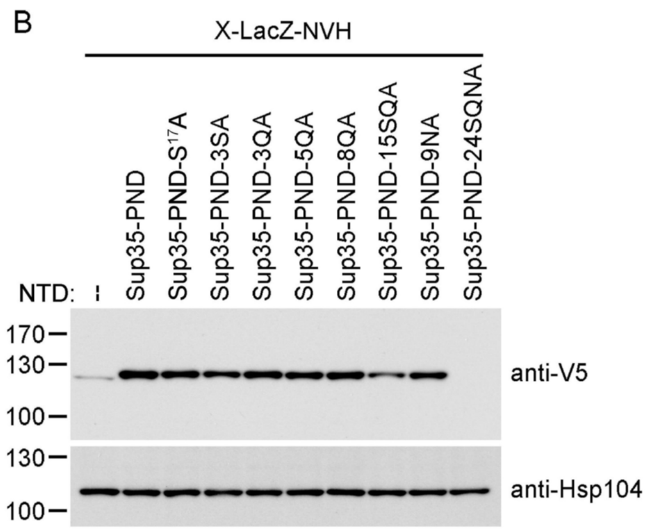
B
X-LacZ-NVH
NTD:
Sup35-PND
Sup35-PND-S17A
Sup35-PND-3SA
Sup35-PND-3QA
Sup35-PND-5QA
Sup35-PND-8QA
Sup35-PND-15SQA
Sup35-PND-9NA
Sup35-PND-24SQNA
170
130
100
anti-V5
130
100
anti-Hsp104

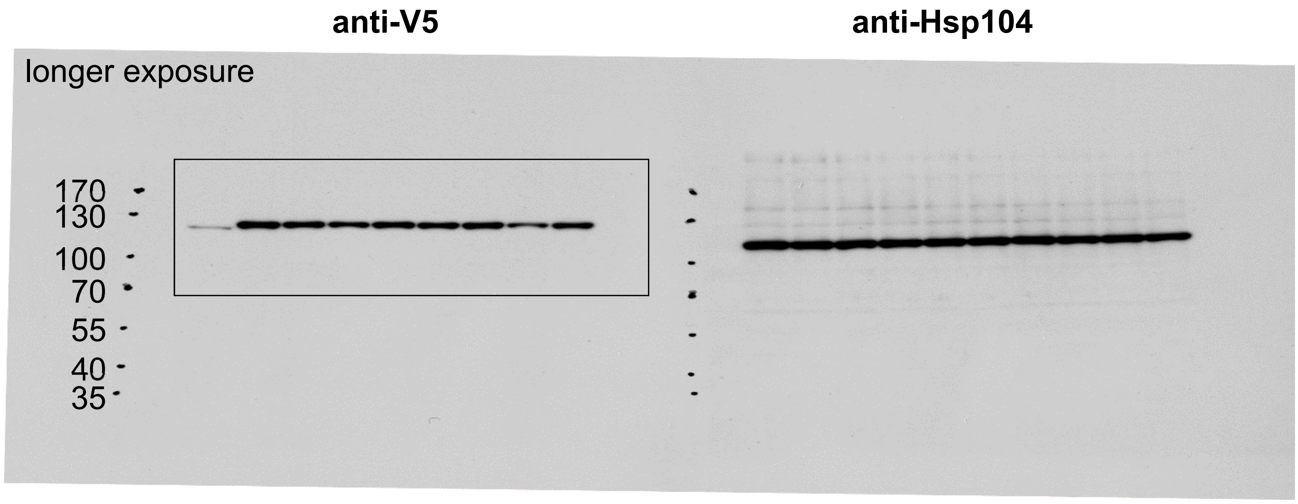
anti-V5
anti-Hsp104
longer exposure
170
130
100
70
55
40
35

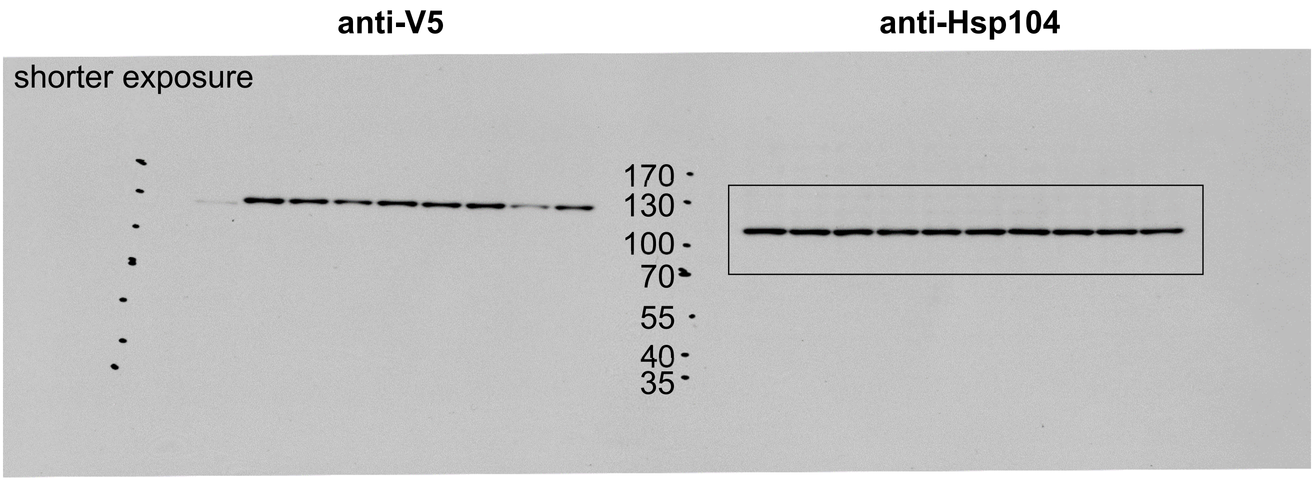
anti-V5
anti-Hsp104
shorter exposure
170
130
100
70
55
40
35

Figure 5

C

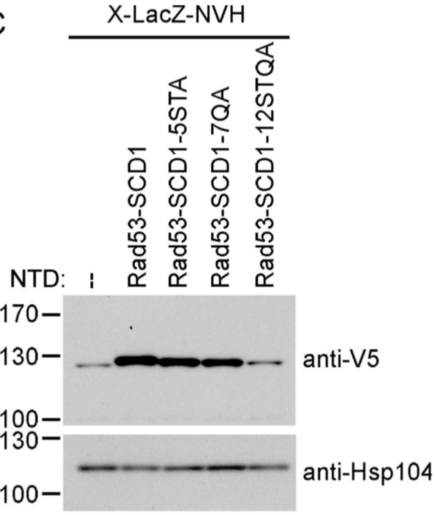

anti-V5

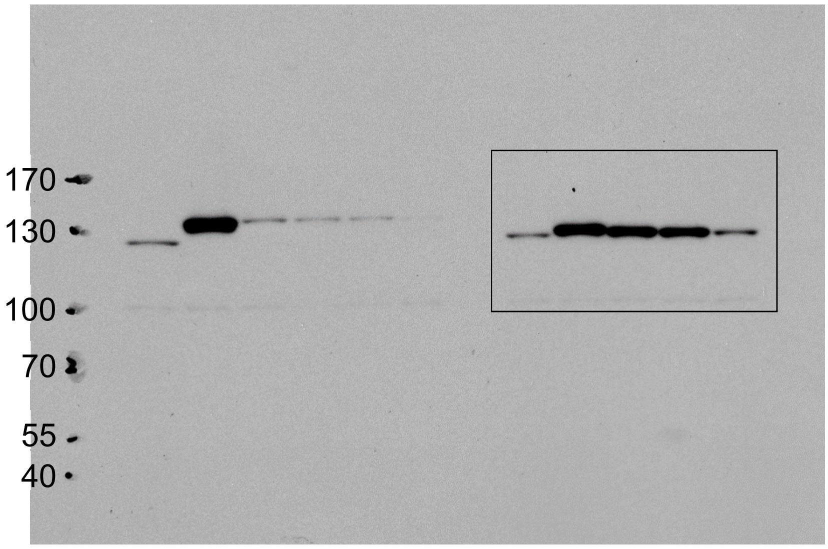

anti-Hsp104

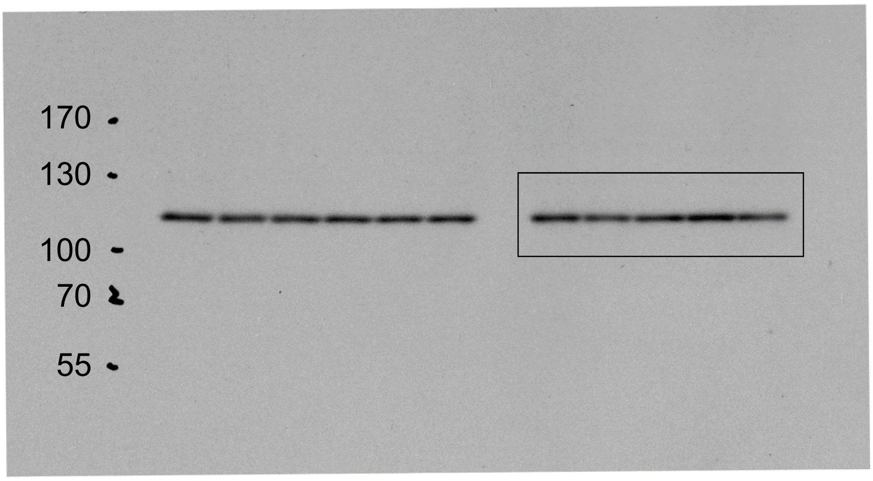

Supplement: Figure 5—source data 1. [file elife-91405-fig5-data1.zip › Figure5/Figure5_source_data_labelled.pdf]
