# Supplementary material for: Noncanonical usage of stop codons in ciliates expands proteins with structurally flexible Q-rich motifs
Source: eLife. 2024 Feb 23;12:RP91405. doi: 10.7554/eLife.91405 (PMC10942620; doi:10.7554/eLife.91405)
Supplement: Supplementary file 1. [file elife-91405-supp1.docx]

Supplementary file 1a. Plasmids used in this study

| Plasmid number | Description |
| --- | --- |
| pTFW2592 | pYC2/NT-C, The mock control. |
| pTFW9957 | *P_RAD51_-LacZ-NVH* |
| pTFW9958 | *P_RAD51_-RAD51-NTD(1-66 a.a.)- LacZ-NVH* |
| pTFW9962 | *P_RAD51_-RAD53-SCD1(1-29 a.a.)-LacZ-NVH* |
| pTFW9963 | *P_RAD51_-HOP1-SCD(258-324 a.a.)-LacZ-NVH* |
| pTFW10027 | *P_RAD51_-SML1-NTD(1-27 a.a.)-LacZ-NVH* |
| pTFW10028 | *P_RAD51_-SML1-NTD(1-50 a.a.)-LacZ-NVH* |
| pTFW10005 | *P_RAD51_-SUP35-PND(1-39 a.a.)-LacZ-NVH* |
| pTFW10221 | *P_RAD51_-SUP35-PFD(1-114 a.a.)-LacZ-NVH* |
| pTFW10169 | *P_RAD51_-NEW1-NPD(1-156 a.a.)-LacZ-NVH* |
| pTFW10177 | *P_RAD51_-URE2-UPD(1-91 a.a.)-LacZ-NVH* |
| pTFW9959 | *P_RAD51_-rad51-NTD-3SA-LacZ-NVH* |
| pTFW10095 | *P_RAD51_-rad51-NTD-8STA-LacZ-NVH* |
| pTFW10096 | *P_RAD51_-rad51-NTD-11STA-LacZ-NVH* |
| pTFW9993 | *P_RAD51_-rad51-NTD-6SQA-LacZ-NVH* |
| pTFW9994 | *P_RAD51_-rad51-NTD-9SQA-LacZ-NVH* |
| pTFW9995 | *P_RAD51_-rad51-NTD-12SQA-LacZ-NVH* |
| pTFW10010 | *P_RAD51_-rad51-NTD-3QA -LacZ-NVH* |
| pTFW10254 | *P_RAD51_-rad51-NTD-9QA-LacZ-NVH* |
| pTFW10255 | *P_RAD51_-rad51-NTD-4NA-LacZ-NVH* |
| pTFW10256 | *P_RAD51_-rad51-NTD-13QNA-LacZ-NVH* |
| pTFW10000 | *P_RAD51_-rad53-SCD1-5STA-LacZ-NVH* |
| pTFW10001 | *P_RAD51_-rad53-SCD1-7QA-LacZ-NVH* |
| pTFW10002 | *P_RAD51_-rad53-SCD1-12STQA-LacZ-NVH* |
| pTFW10034 | *P_RAD51_-sup35-PND-1SA-LacZ-NVH* |
| pTFW10101 | *P_RAD51_-sup35-PND-3SA-LacZ-NVH* |
| pTFW10081 | *P_RAD51_-sup35-PND-3QA-LacZ-NVH* |
| pTFW10080 | *P_RAD51_-sup35-PND-5QA-LacZ-NVH* |
| pTFW10082 | *P_RAD51_-sup35-PND-8QA-LacZ-NVH* |
| pTFW10141 | *P_RAD51_-sup35-PND-15SQA-LacZ-NVH* |
| pTFW10142 | *P_RAD51_-sup35-PND-9NA-LacZ-NVH* |
| pTFW10143 | *P_RAD51_-sup35-PND-24SQNA-LacZ-NVH* |
| pTFW9974 | *P_RAD51_-GFP-NVH* |
| pTFW9975 | *P_RAD51_-RAD53-SCD1-GFP-NVH* |
| pTFW9976 | *P_RAD51_-GST-NVH* |
| pTFW9977 | *P_RAD51_-RAD53-SCD1-GST-NVH* |
| pTFW10003 | *P_RAD51_- GSTnd-NVH* |
| pTFW10004 | *P_RAD51_-RAD53-SCD1-GSTnd-NVH* |

Supplementary file 1b. *S. cerevisiae* strains used in this study

| Name | Genotype |
| --- | --- |
| WHY13008 | *MATa, ho, leu2, ura3, his4-X::LEU2-(NgoMIV;+ori)-URA3, ERG1(SpeI), RAD51::hphMX4* |
| WHY13283 | *MATa, ho, leu2, ura3, his4-X::LEU2-(NgoMIV;+ori)-URA3, ERG1(SpeI), rad51*Δ*::hphMX4* |
| WHY13416 | *MATa, ho, leu2, ura3, his4-X::LEU2-(NgoMIV;+ori)-URA3, ERG1(SpeI), rad51*Δ*N::hphMX4* |
| WHY13744 | *MATα, ho::LYS2, leu2, ura3, lys2, HIS4::LEU2-(BamHI;+ori), Erg1(SalI), dmc1::kanMX4, RAD53^SCD1^-rad51*Δ*N::hphMX4* |
| WHY13743 | *MATa, ho, leu2, ura3, his4-X::LEU2-(NgoMIV;+ori)-URA3, ERG1(SpeI), dmc1::kanMX4, rad53^SCD1-5STA^-rad51*Δ*N::hphMX4* |
| WHY13741 | *MATα, ho::LYS2, leu2, ura3, lys2, HIS4::LEU2-(BamHI;+ori), ERG1(SalI), SUP35^PND^-rad51*Δ*N::hphMX4* |
| WHY10271 | *MATa, ho::hisG, lys2, leu2::hisG, arg4-nsp, ura3* |
| WHY13970 | *MATa his3*Δ*1, leu2*Δ*0, met15*Δ*0, ura3*Δ*0* |
| WHY13785 | *MATa his3*Δ*1, leu2*Δ*0, met15*Δ*0, ura3*Δ*0, hsp104*:*:kanMX4* |
| WHY14126 | *MATa his3*Δ*1, leu2*Δ*0, met15*Δ*0, ura3*Δ*0, new1*:*:kanMX4* |
| WHY14129 | *MATa his3*Δ*1, leu2*Δ*0, met15*Δ*0, ura3*Δ*0, doa4*:*:kanMX4* |
| WHY14227 | *MATa his3*Δ*1, leu2*Δ*0, met15*Δ*0, ura3*Δ*0, doa1*:*:kanMX4* |
| WHY13989 | *MATa his3*Δ*1, leu2*Δ*0, met15*Δ*0, ura3*Δ*0, san1*:*:kanMX4* |
| WHY14132 | *MATa his3*Δ*1, leu2*Δ*0, met15*Δ*0, ura3*Δ*0, oaz1*:*:kanMX4* |

Supplementary file 1c. The oligonucleotide primers used for g-qPCR and RT-qPCR

| Gene | Primer Polarity | Name | Sequence |
| --- | --- | --- | --- |
| *ACT1* | Sense | PA11240 | 5’-CCACCACTGCTGAAAGAGAAATTGT-3’ |
|  | Antisense | PA11241 | 5’-CTTGACCATCTGGAAGTTCGTAGGA-3’ |
| *LacZ* | Sense | PA11246 | 5’-CCGCCGTTTGTTCCCACGGA-3’ |
|  | Antisense | PA11247 | 5’-CCATCACCGCGAGGCGGTTT-3’ |

Supplementary file 1d. The JavaScript software programs used in this study are available at Github (https://github.com/tfwangasimb/AS-Q-rich-motif)

| Program name | Purpose |
| --- | --- |
| AS-aa-content | Determination of proteome-wide contents of 20 different amino acids |
| AS-codon-usage | Determination of proteome-wide frequency of 64 genetic codons |
| AS-Finder-SCD | Proteome-wide search of the SCD motifs |
| AS-Finder-7polyX | Proteome-wide search of the 7 different types of polyX motifs |
| AS-Xcontent-7polyX | Determination of the ratios of the overall number of X residues for each of the seven polyX motifs relative to those in the entire proteome of each species |
| AS-GOfuncR-FWER | Statistical analysis of GO enrichment datasets |
